# Paired oral clinical specimens reveal the underlying ecology supporting the emergence of inflammophilic microbiome communities

**DOI:** 10.1128/spectrum.01550-26

**Published:** 2026-09-14

**Authors:** Madeline Krieger, Kristopher A. Kerns, Elizabeth A. Palmer, Jeffrey S. McLean, Jens Kreth, Galip Gürkan Yardımcı, Justin L. Merritt

**Affiliations:** 1 Department of Biomaterial and Biomedical Sciences, School of Dentistry, Oregon Health & Science University (OHSU) 212162, Portland, Oregon, USA; 2 Cancer Early Detection Advanced Research Center, Knight Cancer Institute, Oregon Health & Science University (OHSU) 643695, Portland, Oregon, USA; 3 Department of Periodontics, University of Washington 7284, Seattle, Washington, USA; 4 Division of Pediatric Dentistry, School of Dentistry, Oregon Health & Science University (OHSU) 212162, Portland, Oregon, USA; 5 Department of Molecular Microbiology and Immunology, Oregon Health & Science University (OHSU) 547642, Portland, Oregon, USA; 6 Division of Oncological Sciences, Oregon Health & Science University (OHSU) 6684, Portland, Oregon, USA; The Ohio State University College of Dentistry, Columbus, Ohio, USA

**Keywords:** oral microbiome, microbiome, oral dysbiosis, odontogenic abscess

## Abstract

Across human mucosal sites, dysbiotic inflammatory diseases are characterized by compositional shifts in the resident microbiota, in which commensal-dominated communities give way to pathobiont-enriched communities where “inflammophilic” species often predominate. Effective treatments for these complex polymicrobial infections remain limited, in part, because the ecological mechanisms driving their emergence and persistence are still poorly understood. To address this gap, we analyzed a unique cohort of pediatric patient-matched, disease-free dental plaque and odontogenic abscess specimens, providing a clinically relevant model to examine microbiome transitions from commensal to inflammophilic states. Using complementary community ecology modeling approaches and inferred metagenomic analyses, we identified microbial taxa and functional programs associated with inflammatory selective pressure and dysbiotic community emergence. Dental plaque communities are characterized by anabolic metabolic processes and carbohydrate-derived ATP generation, whereas abscess microbiomes are highly biased for catabolic metabolism, amino acid-derived ATP generation, and antimicrobial resistance. The results suggest that abscess communities are much less reliant upon interspecies metabolic complementation compared to dental plaque communities, which would imply an obligate dependence upon host inflammatory responses to provide the key metabolites required for growth. These findings support a model in which an inflammophilic community ecology is largely the net result of a combination of enhanced resistance to innate immunity and compatibility with the inflammatory nutrient environment. By defining the metabolic requirements and selective pressures governing these dysbiotic transitions, it may be possible to suppress inflammatory dysbiotic diseases using ecologically focused therapeutic strategies that exploit the limited biosynthetic capacity of commensal depleted inflammophilic communities.

**IMPORTANCE:**

Dysbiotic inflammatory diseases are frequently sustained by complex microbial community interactions, but the ecological processes involved remain poorly understood. In this study, we leveraged pediatric patient-matched, disease-free dental plaque and odontogenic abscess clinical specimens to examine how oral microbial communities shift from health-associated to inflammation-associated states. We found that abscess microbiomes are enriched for features consistent with adaptation to inflammatory environments, including antimicrobial resistance, catabolic metabolism, and utilization of host-derived nutrients. These findings support a model in which host inflammation functions as a selective ecological pressure, favoring the establishment of metabolically specialized, inflammophilic microbial communities. By defining the ecological and functional features that distinguish abscess-associated communities from disease-free plaque microbiota, this work provides a framework for understanding inflammatory dysbiosis and for developing ecological strategies to disrupt pathobiont-enriched communities while promoting the restoration of stable, health-associated microbiota.

## INTRODUCTION

Despite major compositional differences among site-specific microbiota, dysbiotic inflammatory diseases occur across all human mucosal surfaces (1). Host-derived inflammatory pressure is associated with the emergence of dysbiotic microbiome communities via poorly defined mechanisms. These communities persist and even thrive despite host antimicrobial inflammatory responses (2). Under the appropriate conditions, this can lead to the positive feedback cycle that characterizes various mucosal chronic inflammatory diseases, whereby inflammatory responses appear to sustain these complex polymicrobial infections instead of resolving them. While there is strong evidence that such diseases are rooted in the underlying ecology of the dysbiotic microbial communities (3), many fundamental questions remain about disease etiology, and efficacious treatment strategies remain elusive.

Like other mucosal sites, the oral cavity is subject to a variety of polymicrobial dysbiotic inflammatory diseases, with periodontitis and odontogenic abscesses being among the most common (2, 4). The oral cavity supports a complex microbial ecosystem that is second only to the gastrointestinal tract in measures of biodiversity (5). Oral biofilms (i.e., dental plaque) that form on the dentition and surrounding epithelial surfaces are primarily composed of commensal species during eubiosis. However, oral microbiome composition changes dramatically during diseased, dysbiotic conditions (6–11). Human odontogenic abscesses are an intriguing clinical platform to investigate the ecological mechanisms supporting inflammatory dysbiosis, as the abscess communities are initially seeded by the commensal dental plaque microbiota (12, 13) but undergo a dramatic and rapid compositional transformation as the abscess develops. This transition is thought to be a consequence of host inflammatory selective pressure (14–16), resulting in communities that become increasingly dominated by a variety of “inflammophilic” microbiota (2, 17), such as species of *Fusobacterium*, *Prevotella*, *Parvimonas*, and others (18–22). While such organisms are commonly detected in oral abscess studies (4, 23), variability in the results has challenged the discernment of the broader ecological characteristics of inflammophilic microbiomes. Much of this variability can be attributed to the myriad of factors influencing community-level fluctuations in the microbiomes of different individuals, such as diet, lifestyle, genetics, and environment (24–27). Consequently, we assembled a unique cohort of paired disease-free dental plaque and odontogenic abscess clinical specimens concurrently sampled from single individuals to mitigate the impact of these confounders. Additionally, the sequestration and inherent physical separation of abscess and dental plaque communities further alleviate the negative effects of clinical specimen cross contamination during sampling procedures. From this cohort, we could establish a patient-specific baseline community composition in each dental plaque specimen to examine how its particular community ecology has evolved within the abscess due to inflammatory selective pressure from the host. Our results suggest abscess communities lose much of the metabolic interdependencies that characterize free living commensal microbiomes, implying that inflammatory dysbiotic communities may be vulnerable to therapeutic interventions that exploit their reliance upon host-derived metabolites for persistence.

## RESULTS

### Dental plaque and abscess community compositions are divergent

**Fig 1:**
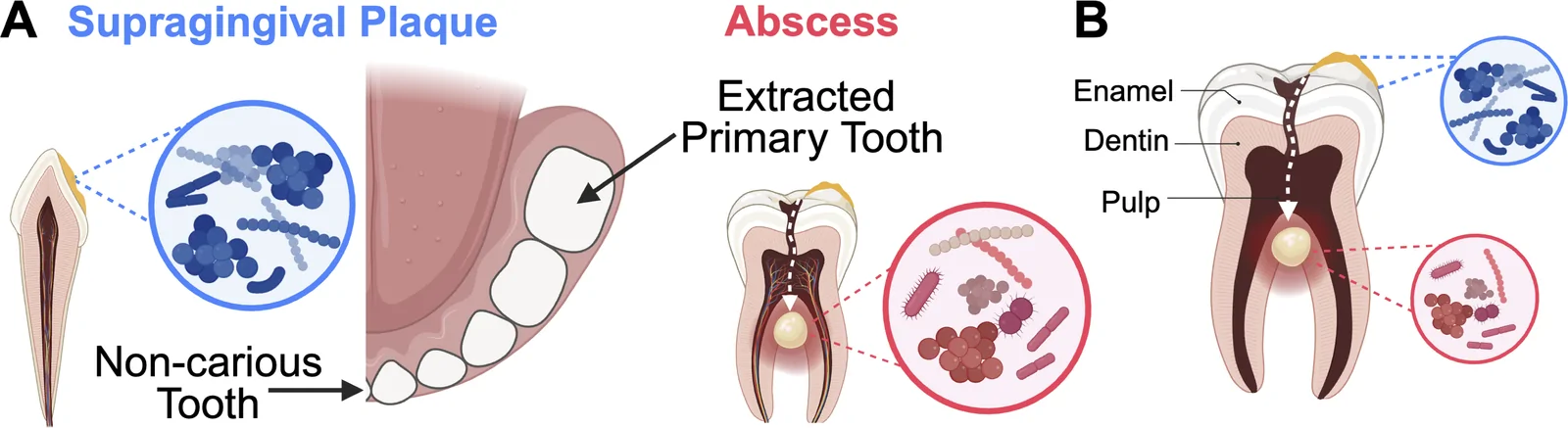
Patient sampling and the ecology of oral abscess formation. (**A**) The cohort was constructed from patient-matched specimens taken from the supragingival plaque of non-carious primary canines and the abscess material of an extracted primary molar. (**B**) Caries development begins with demineralization of the enamel surface. If left untreated, the lesion can progress into a deep caries lesion that approaches the tooth pulp chamber. Once the caries lesion has breached the pulp chamber, a direct pathway is created between the oral cavity and the normally sterile tooth pulp, allowing oral microbiota from dental plaque to invade through the caries lesion. Colonization of the pulp chamber initiates an infection that can spread through the root canal to the tooth apex or root furcation, resulting in apical or furcal (pictured) abscess formation.

Our cohort of patient-matched dental plaque and abscess clinical specimens was used to directly investigate the ecological influence of host inflammatory pressure upon the oral microbiota. Odontogenic abscesses are directly seeded by extant dental plaque communities (12, 13) that become sequestered and isolated from external influences once the host assembles walled abscesses far below the gum line (Fig. 1). This unique feature of tooth abscess physiology also limits the common problem of specimen cross-contamination with the oral microbiota during clinical sampling, as abscess communities are only revealed following tooth extraction and remain physically separated from surrounding dental plaque throughout the procedure. To investigate the differences between dental plaque and odontogenic abscess communities, we performed 16S rRNA V3V4 gene sequencing of 25 paired, patient-matched plaque and abscess specimens generated from regularly scheduled clinical interventions at the Oregon Health and Science University pediatric dental clinic.

**Fig 2:**
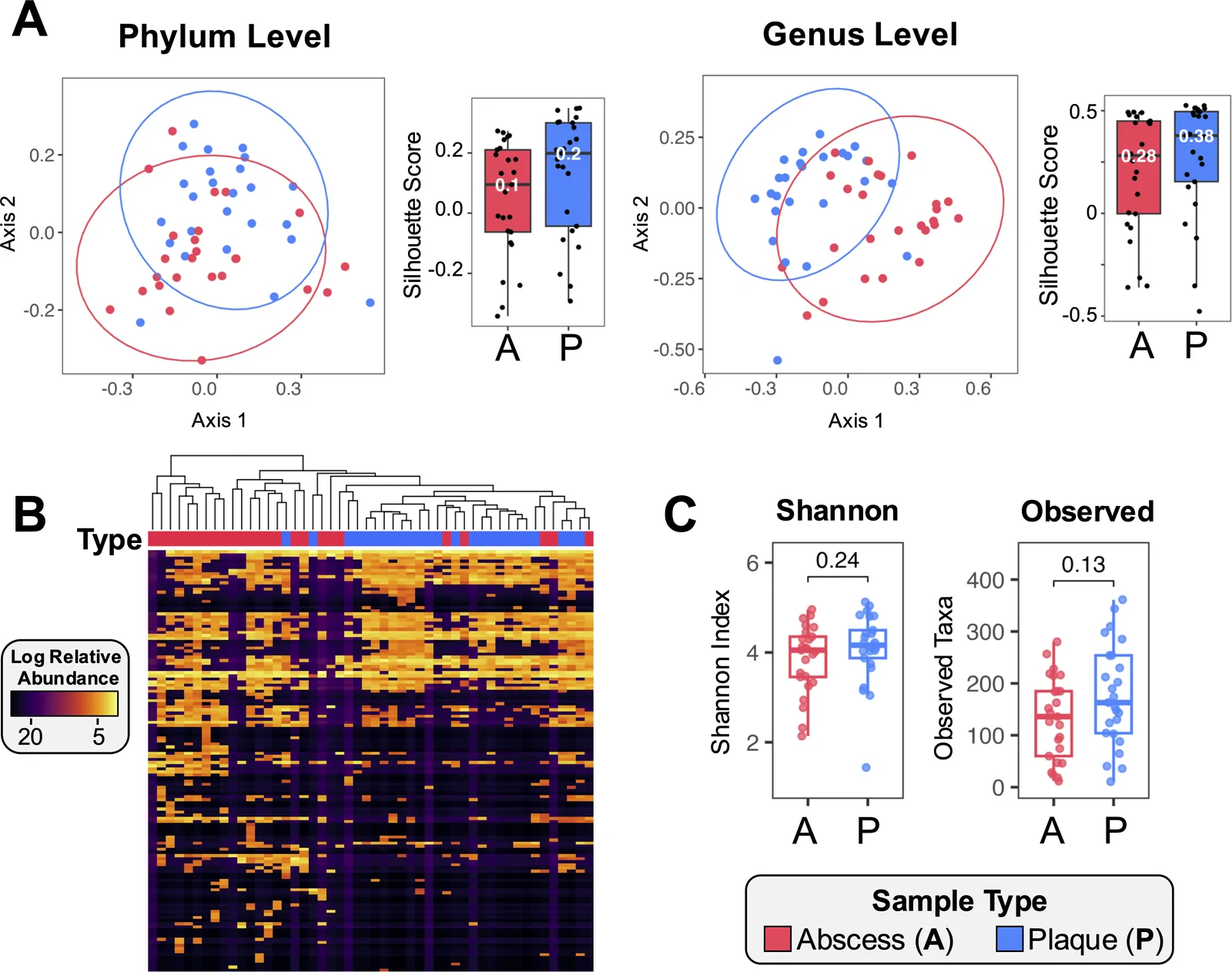
Ecological composition of plaque and abscess communities. (**A**) Principal coordinate analysis (PCoA) of relative abundance values using Bray–Curtis distance at the phylum (left) and genus (right) levels. Silhouette scores are shown alongside each PCoA plot. (**B**) Hierarchical clustering (Pearson correlation) of log-transformed relative abundances of genera separates plaque and abscess communities with some overlap. (**C**) Alpha diversity measured by Shannon and Observed richness indices, with *P*-values from Wilcoxon Signed-rank tests are displayed above the two groups.

To assess between-sample variation, we profiled beta diversity using Principal Coordinate Analysis (PCoA) (Fig. 2A). The first ordination axis captured true biological variation rather than sequencing artifacts, supported by a minimal correlation with library size (Spearman *ρ* = 0.18 at the phylum level and –0.25 at the genus level). We further measured the median silhouette width for each community type to assess the ability of PCoA to distinguish plaque and abscess communities using a metric we refer to as a silhouette score. Results of these PCoA analyses indicated that community composition can distinguish between plaque and abscess specimens, especially at the genus and species levels (Fig. 2A and Fig. S1A). Additional paired PERMANOVA analysis of the two communities reflects the significant compositional differences between plaque and abscess samples at all three taxonomic levels: Species (*R*² = 0.10, *P* = 0.001), Genus (*R*² = 0.14, *P* = 0.001), and Phylum (*R*² = 0.10, *P* = 0.001). Betadisper analysis showed significant differences in multivariate dispersion between groups at the Species (*P* = 0.012) and Genus (*P* = 0.017) levels, indicating that compositional separation at these levels may partly reflect unequal within-group heterogeneity rather than a purely compositional shift. At the Phylum level, however, dispersion did not differ significantly between groups (*P* = 0.252), supporting a cleaner interpretation that the observed separation reflects a true compositional shift rather than a dispersion artifact.

Hierarchical clustering of genus and species level relative abundances using Pearson correlation similarly revealed clear distinctions between plaque and abscess communities (Fig. 2B and Fig. S1B). We next compared within-sample diversity using Shannon and Observed richness metrics of the amplicon sequence variants (ASVs) present in each sample (Fig. 2C). Neither metric was significantly different between the two communities, suggesting that they are similar in both the total number of unique taxa and the evenness of taxa composition in the specimens. We noted a weak trend toward greater biodiversity in the plaque specimens, but it did not reach statistical significance (observed alpha diversity, Wilcoxon Signed-rank *P* = 0.13) (Fig. 2C, right).

### Comparison of taxa enrichment using multiple differential abundance metrics

**Fig 3:**
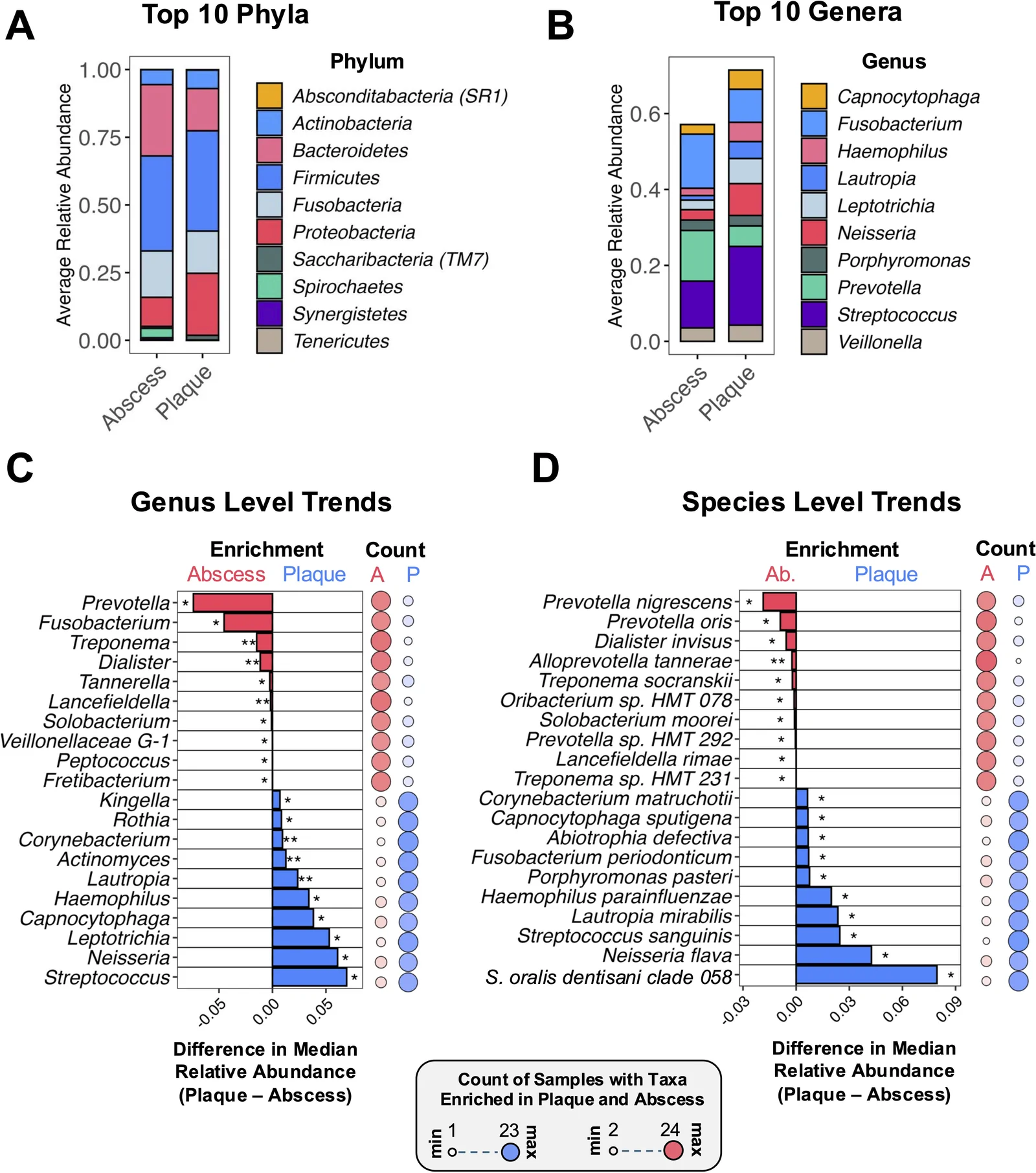
The distribution of taxa within oral plaque and abscess specimens. Average relative abundances of the (**A**) top 10 phyla and (**B**) top 10 genera in plaque and abscess specimens. (**C**) Genus- and (**D**) species-level enrichment trends calculated by the whole-group difference in median average abundance. The bar plot (left) is ranked by taxa with the greatest differences between plaque and abscess abundance. The bubble plot (right) signifies the number of samples that have higher relative abundance values of each taxon in the abscess (red) or plaque (blue) communities. Asterisks indicate BH-adjusted *P*-values from the paired Wilcoxon signed-rank test (*P < 0.05, **P < 0.01, ***P < 0.001).

After confirming the presence of distinct community compositions in plaque and abscess specimens, we were next interested to compare the differential abundances of the specific taxa in the two communities. The overall composition of each community is shown in Fig. 3A and B. At the phylum level, plaque and abscess communities are broadly similar (Fig. 3A). However, at the genus level, compositional distinctions become clearly discernable (Fig. 3B). The top 20 genera with the largest significant differences between median values of the two groups were determined using a paired Wilcoxon Signed-rank test (Benjamini-Hochberg false discovery rate corrected *P*-value < 0.05) (Fig. 3C; Table S1), which is a straightforward statistical assessment useful for identifying robust, consistent changes across samples. To further confirm that the observed trends were not attributable to major outliers, we also tallied the number of specimens exhibiting enrichment of each genus in the plaque and abscess specimens. These counts are presented as a bubble plot together with the respective enrichment results (Fig. 3C; Table S2). For example, genera mainly associated with oral health, such as *Neisseria*, *Lautropia*, *Actinomyces,* and *Rothia* (28), were strongly associated with the plaque specimens, whereas the *Prevotella*, *Treponema*, and *Dialister* (29, 30) genera stratified with the abscesses, in agreement with the prominent inflammophilic species they contain (31, 32). We also further resolved these data to the species level (Fig. 3D; Table S3). The common health-associated commensal species *Streptococcus oralis* subsp. *dentisani*, *Neisseria flava*, *Streptococcus sanguinis* (33), *Lautropia mirabilis*, and *Haemophilus parainfluenzae* (34) all dominated the plaque specimens. Conversely, the oral pathobionts *Prevotella nigrescens*, *Prevotella oris*, *Dialister invisus*, and *Alloprevotella tannerae* (31) were strongly associated with the abscesses.

**Fig 4:**
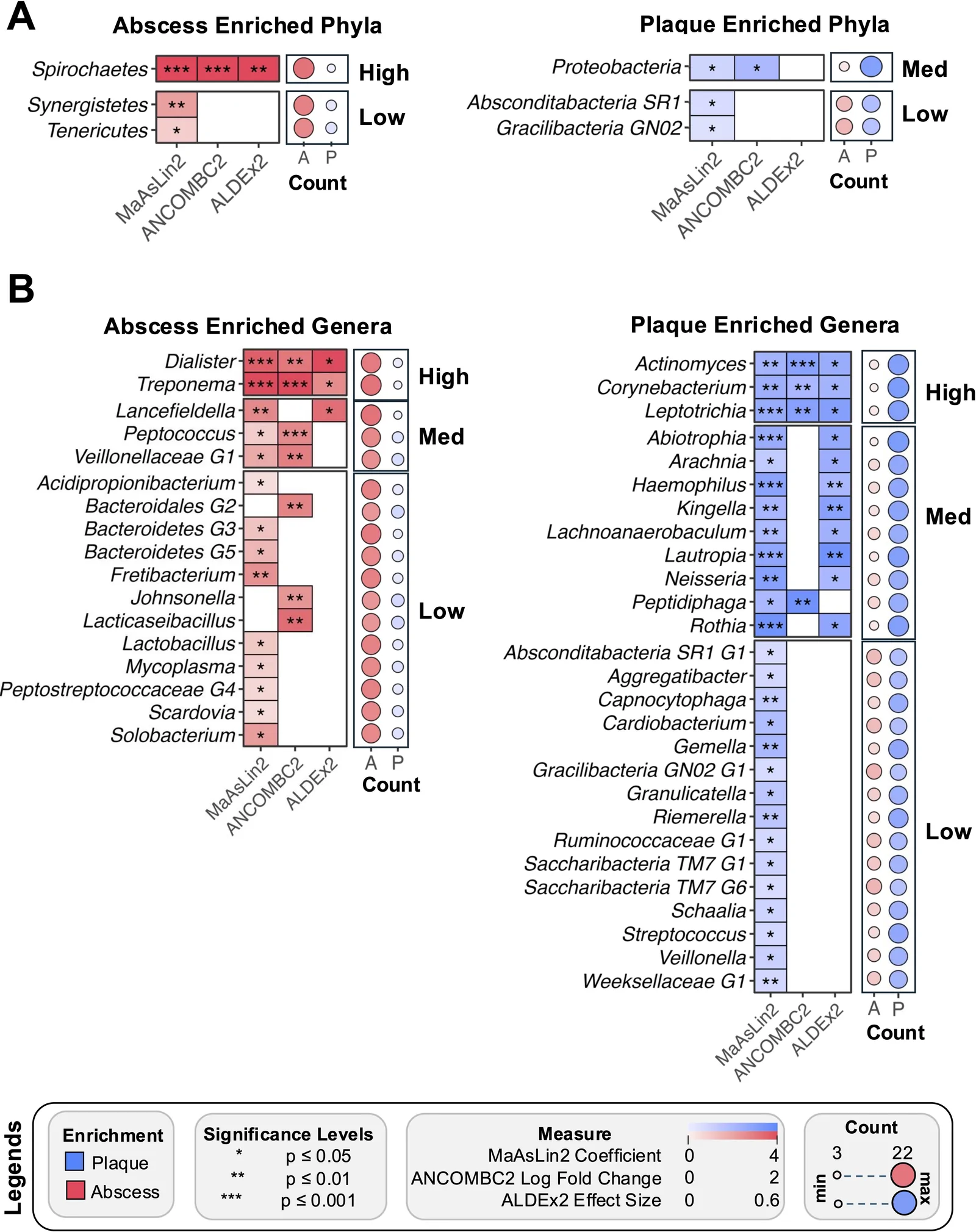
Differentially abundant phylum- and genus-level taxa in plaque and abscess communities. Results from three differential abundance methods (MaAsLin2, ALDEx2, ANCOMBC2). Significant enrichment of a taxon via all three methods resulted in its classification as high confidence, significant enrichment in two methods resulted in a medium confidence classification, and significance from a single method was classified as low confidence. The bubble plot to the right of each plot signifies the number of samples exhibiting enrichment in the abscess (red) or plaque (blue) communities. (**A**) Phylum-level results. (**B**) Genus-level results.

Given the significant differences observed from the paired Wilcoxon Signed-rank test, we next analyzed the sequence results using differential abundance analytic approaches specifically tailored for microbiome biomarker discovery. Due to the disparate results commonly reported from studies using different differential abundance analysis methods (35, 36), we analyzed the plaque and abscess sequence data using three commonly employed approaches: Microbiome Multivariable Associations with Linear Models (MaAsLin2) (37), Analysis of Compositions of Microbiomes with Bias Correction (ANCOMBC2) (38), and Analysis Of Differential Abundance Taking Sample and Scale Variation Into Account (ALDEx2) (39). MaAsLin2 and ALDEx2 use generalized linear models, with MaAsLin2 allowing incorporation of metadata covariates. ANCOMBC2 uses a mixed-effects framework with covariate consideration. Accordingly, each of these differential abundance algorithms resulted in slightly different outcomes (Table S4). Thus, enriched taxa identified by all three methods were labeled as high confidence, whereas medium confidence taxa were identified by two methods, and low confidence taxa were unique to a single algorithm. At the phylum level, we identified one high-confidence taxon, *Spirochetes*, which was enriched in the abscess. One medium-confidence phylum, *Proteobacteria*, was enriched in the plaque, (Fig. 4A), along with four low confidence enriched phylum: *Synergistetes* and *Tenericutes* in the abscess and the relatively understudied phyla *Absconditabacteria* SR1 and *Gracilibacteria* GN02 in the plaque. At the genus level, five high confidence taxa were identified: *Leptotrichia*, *Corynebacterium*, and *Actinomyces* for dental plaque specimens and *Dialister* and *Treponema* for abscesses (Fig. 4B). As in Fig. 3C and D, a count of samples with enrichment of each taxon in the specimens is shown to the right of each plot, with specific count values presented in Table S5. Dot plots for the relative abundance/total sum scaling (TSS) values of these high and medium confidence genera indicate the results were not driven by outliers in the data (Fig. S2A and B).

**Fig 5:**
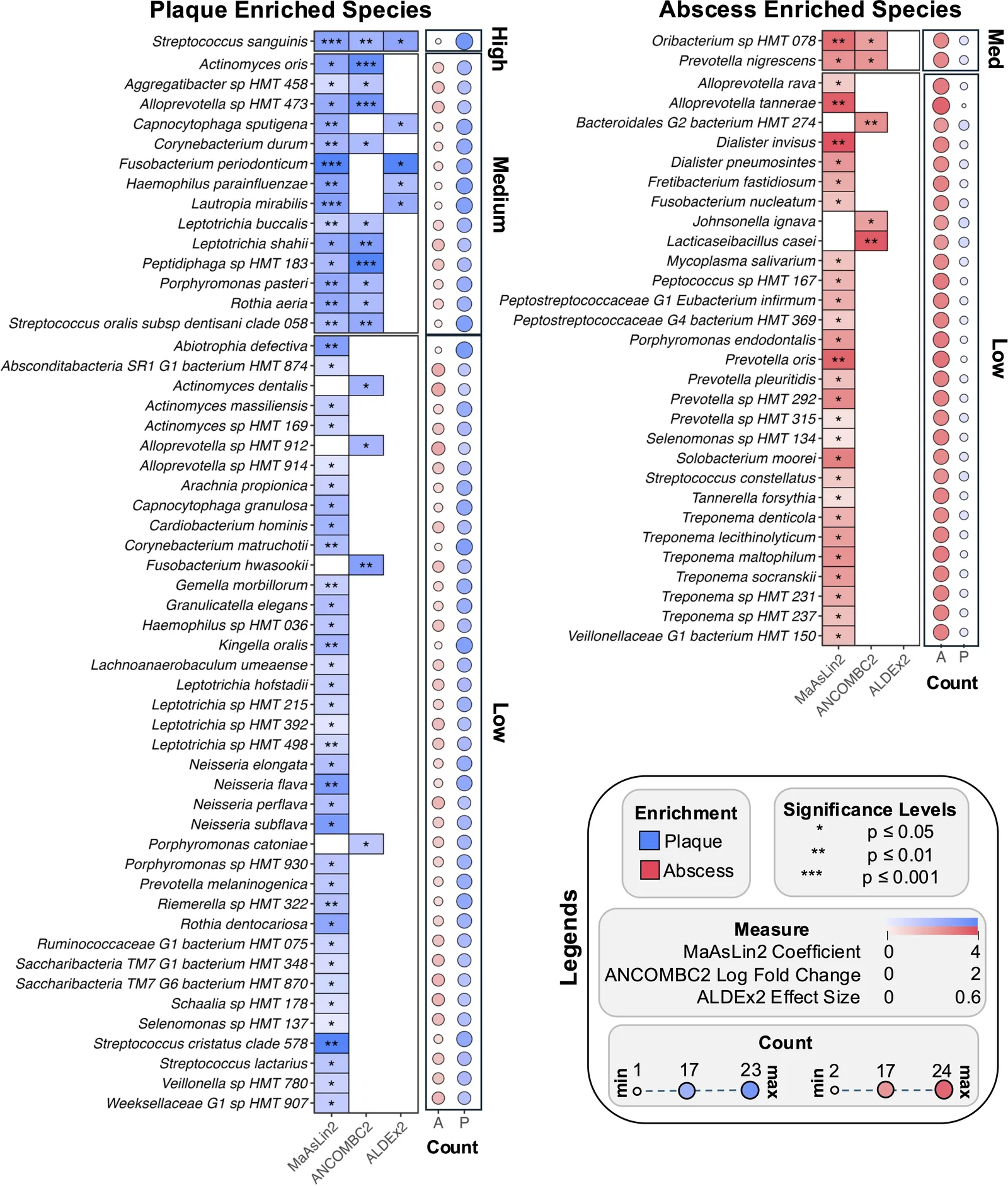
Differentially abundant species in plaque and abscess communities. Results from three differential abundance methods (MaAsLin2, ALDEx2, ANCOMBC2). Significant enrichment of a taxon via all three methods resulted in its classification as high confidence, significant enrichment in two methods resulted in a medium confidence classification, and significance from a single method was classified as low confidence. The bubble plot to the right of each plot signifies the number of samples exhibiting enrichment in the abscess (red) or plaque (blue) communities.

When examining species-level resolution (Fig. 5), only the oral commensal plaque-enriched species *Streptococcus sanguinis* species met our stringent threshold for high confidence enrichment. In the plaque, 14 species were enriched with medium confidence and included *Rothia aeria*, *Corynebacterium durum*, and *Porphyromonas pasteri* (40). The unclassified taxon *Oribacterium HMT 078* and the well-studied *Prevotella nigrescens* (41) were enriched with medium confidence in the abscess. It is worth noting that we detected a weak enrichment of *Fusobacterium nucleatum* in the abscess (Fig. 5) that was not captured by the genus-level differential abundance analysis methods (Fig. 4) despite known associations of *F. nucleatum* with oral inflammatory diseases (42). Intrigued, we further confirmed these results using TSS and centered log ratio (CLR) transformations to visualize the distribution of fusobacteria within the plaque and abscess specimens (Fig. S3). A recent subspecies-level analysis of *F. nucleatum* in oral clinical specimens identified exceptionally biased subspecies-level stratification of *F. nucleatum* within patient-matched health vs disease specimens (43). Thus, *F. nucleatum* data would likely require sequence methodologies that support resolution to the subspecies level to discern plaque- and abscess-specific enrichment trends (43, 44). Furthermore, due to the aggregation of *Fusobacterium periodonticum* and *F. nucleatum* in the genus-level results, these data were unable to discern abscess-associated *F. nucleatum* enrichment (Fig. 4). This is a clear illustration of how genus-level data can obscure significant species-level trends in microbiome sequence data.

### Topic modeling reveals community signatures within plaque and abscess communities

While differential abundance analyses identified specific plaque- and abscess-enriched taxa within the specimens, these analyses do not capture broader ecological trends in the communities, which is a key facet defining eubiosis vs dysbiosis. Therefore, we subsequently employed two different machine-learning approaches to identify broader community-level trends in the specimens using both supervised and unsupervised methods.

**Fig 6:**
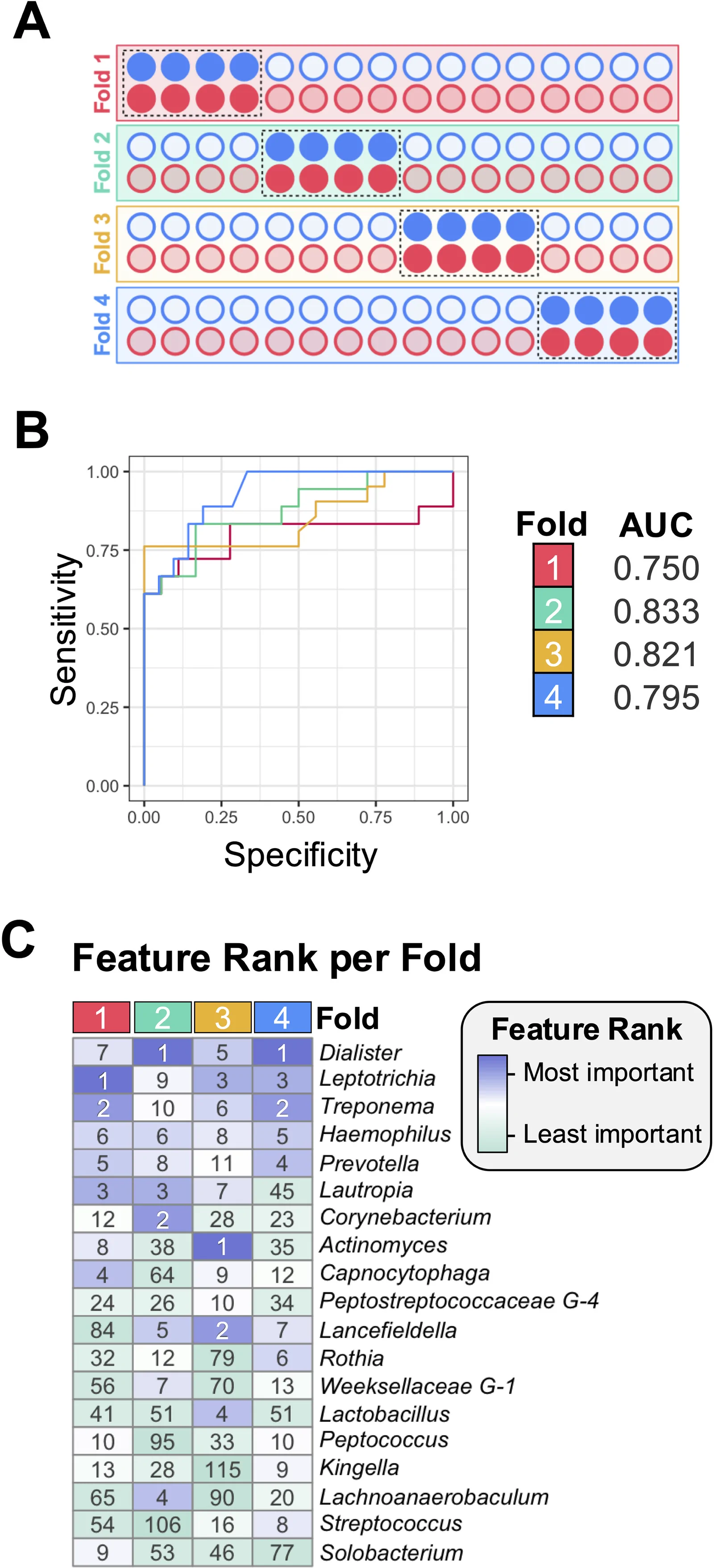
Genus-level community composition predicts clinical specimen type. A random forest classifier was trained on genus-level relative abundances using fourfold cross validation. (**A**) A schematic illustrating how fourfold cross validation was employed to divide the plaque (blue circles) and abscess (red circles) samples into four equal folds, with ¼ of the data (dark colored circles) withheld to test the accuracy of the algorithm trained on the remaining ¾ of the data (light colored circles). (**B**) Receiver operating characteristic (ROC) curves from fourfold cross validation demonstrate strong performance (area under the curve, or AUC values of 0.75–0.83). (**C**) Heatmap of feature importance rankings across folds, with top predictors in purple and lower-ranked features in green. The rank of feature importance is shown inside each cell.

First, we applied random forest classification with k-fold cross-validation. This approach partitions the data set into multiple training and testing folds, enabling the model to learn classification patterns from the training data followed by an evaluation of the performance of the trained model using the remaining test set. This approach is ideally suited to balance bias variance tradeoffs in cohorts with moderate sample sizes; namely, we can train models on a large training set, while assessing the generalizability of the trained models across different folds. We elected to use fourfold cross validation to train random forest models that can distinguish between clinical specimen types in which ¾ of the sequence data were used for training and the remaining ¼ used for analysis (Fig. 6A). Using genus-level compositional data, our model classified specimen type with an area under the ROC curve (AUC) of 0.75–0.83 across the fourfold (Fig. 6B). Although taxa were generally consistent across folds, some variability was observed (Fig. 6C). For example, *Dialister* ranked as the strongest predictor in folds 2 and 4, but fifth and seventh in folds 1 and 3, respectively. Fold 2, which had the highest accuracy (83%), prioritized the genera *Dialister*, *Corynebacterium*, and *Lautropia* as the 1st–3rd most important specimen type predictors. These genera were also significantly enriched in our prior analysis (Fig. 3C; Fig. 4B). Interestingly, the fourth strongest predictor was *Lachnoanaerobaculum,* a genus not previously identified in our differential abundance analyses. Although random forest performed well at the genus level, it was slightly less consistent using species-level data, with accuracy ranging from 55% to 85% (as measured by area under the curve) (Fig. S4A). Much of this variability in community composition could be specifically attributed to fold 1. Despite this, *Streptococcus sanguinis* and *Dialister invisus* remained among the top 16 predictors (based on mean decrease in Gini impurity) between all four folds (Fig. S4B), which is highly consistent with our differential abundance analysis (Fig. 5). Based upon the cohort size and large number of features, some variation across different folds was expected, even for broadly consistent predictors.

**Fig 7:**
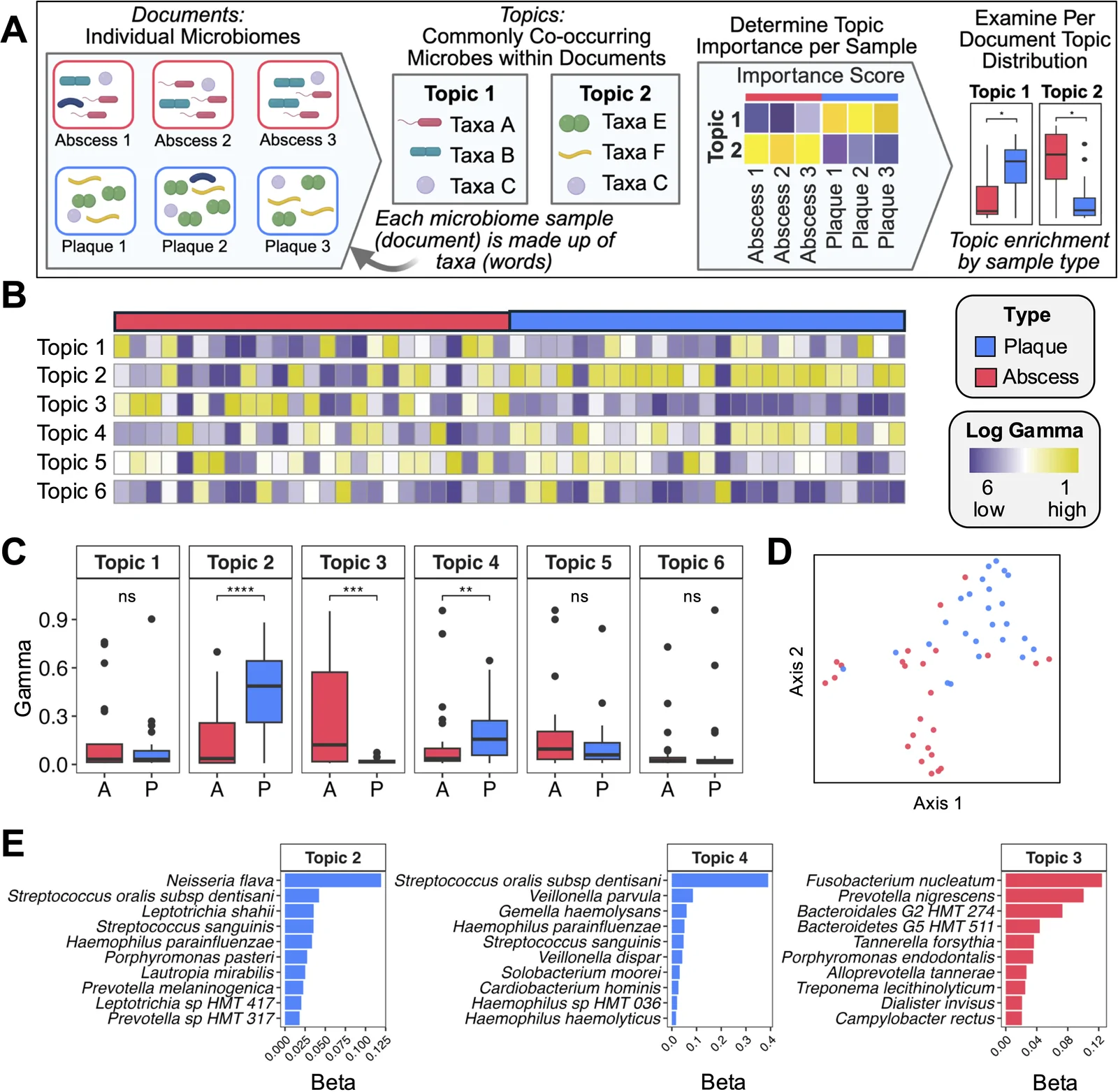
Species-level topic modeling of plaque and abscess specimens. (**A**) An illustration demonstrating how Latent Dirichlet Allocation (LDA) can be applied to microbiome data to elucidate community-level signatures. (**B**) Heatmap of gamma scores across topics. (**C**) Boxplots of sample membership (gamma values), with the results of a Wilcoxon Signed-rank test displayed above (***P < 0.001, *P < 0.05). (**D**) Uniform manifold approximation and projection (UMAP) of gamma scores demonstrate clear plaque–abscess separation. (**E**) The importance (i.e., beta) scores of the top species driving each topic. Only the results from the topics significantly enriched in plaque communities (topics 2 and 4) and abscess communities (topic 3) are shown. The beta scores of the shared topics are shown in Fig. S7.

The observed variability in the species-level random forest results led us to pursue a new analytic approach that we predicted would be ideally suited for microbiome data sets: Latent Dirichlet Allocation (LDA) topic modeling. LDA is an algorithm originally developed for natural language processing to organize text-based documents into underlying themes or topics in an unsupervised fashion, with no prior knowledge of the underlying composition and contents of each topic. LDA has been applied to microbiological data sets in a few prior studies (45–49), but it remains an uncommon analytical approach in the field. Our adaptation of this algorithm employs scaled relative abundance values of microbiome taxa and is summarized in Fig. 7A. In contrast to standard differential abundance methodologies (Fig. 4; Fig. 5), LDA can learn polymicrobial signatures of co-occurring taxa within data sets without each member of the signature being present in all specimens, potentially mitigating the impact of clinical specimen variability while learning signatures that can be broadly shared across clinical specimens in the cohort. After stratifying the microbiome into latent community structures (topics), we next examined the degree of sample membership within each topic (gamma). Using goodness of fit metrics (Fig. S5A), we created a species-level LDA model with six topics (Fig. 7B). A Wilcoxon Signed-rank test yielded a highly significant separation (*P* < 0.001) between several plaque-specific (topics 2 and 4) and abscess-specific (topic 3) topics (Fig. 7C; Fig. S6). Non-significant topics that capture shared signatures of both communities were also identified (topics 1, 5, and 6) (Fig. 7C; Fig. S7). Uniform Manifold Approximation and Projection (UMAP) of gamma values effectively separated plaque and abscess communities (Fig. 7D). The distribution of relative abundances for the top taxa in each topic is presented in Fig. S6, revealing clear enrichment patterns in the plaque- and abscess-dominated topics. The plaque-specific topics are principally driven by the obligately commensal species *Neisseria flava* (topic 2) and *Streptococcus oralis* subsp. *dentisani* (topic 4), whereas *Fusobacterium nucleatum*, *Prevotella nigrescens*, and *Bacteroidales* G2 HMT 274 were the most prominent within the abscess-specific topic 3 (Fig. 7E). Interestingly, topic 3 is also far less biased for a single species compared to the plaque-specific topics 2 and 4. The top drivers of the shared signatures (topics 1, 5, and 6) are shown in Fig. S7.

For comparison, we also repeated the LDA analysis using genus-level data, resulting in three plaque-specific topics (topics 1, 4, and 6), two abscess-specific topics (topics 3 and 5), and two shared topics (topics 2 and 7) (Fig. S5B; Fig. S8A through C and Fig. S9). At this lower level of phylogenetic resolution, LDA analysis still identified key genera driving differential community structures, such as *Neisseria* (plaque) and *Fusobacterium* (abscess) (Fig. S8C). However, there was comparatively less overall distinction between the plaque and abscess specimens, presumably due to the mixture of oral eubiont and pathobiont species found in many genera. For example, the genus *Fusobacterium* appeared as a main driver in both the abscess-specific topic 5 and shared signature topic 2 (Fig. S8 and Fig. S9). This illustrates the flexibility of LDA for microbiome analysis, as the same taxon can appear in multiple topics. Collectively, these results support the utility of topic models for revealing polymicrobial signatures present within complex microbiome communities.

### Biochemical pathway analysis of plaque and abscess communities implicates environmental selection

**Fig 8:**
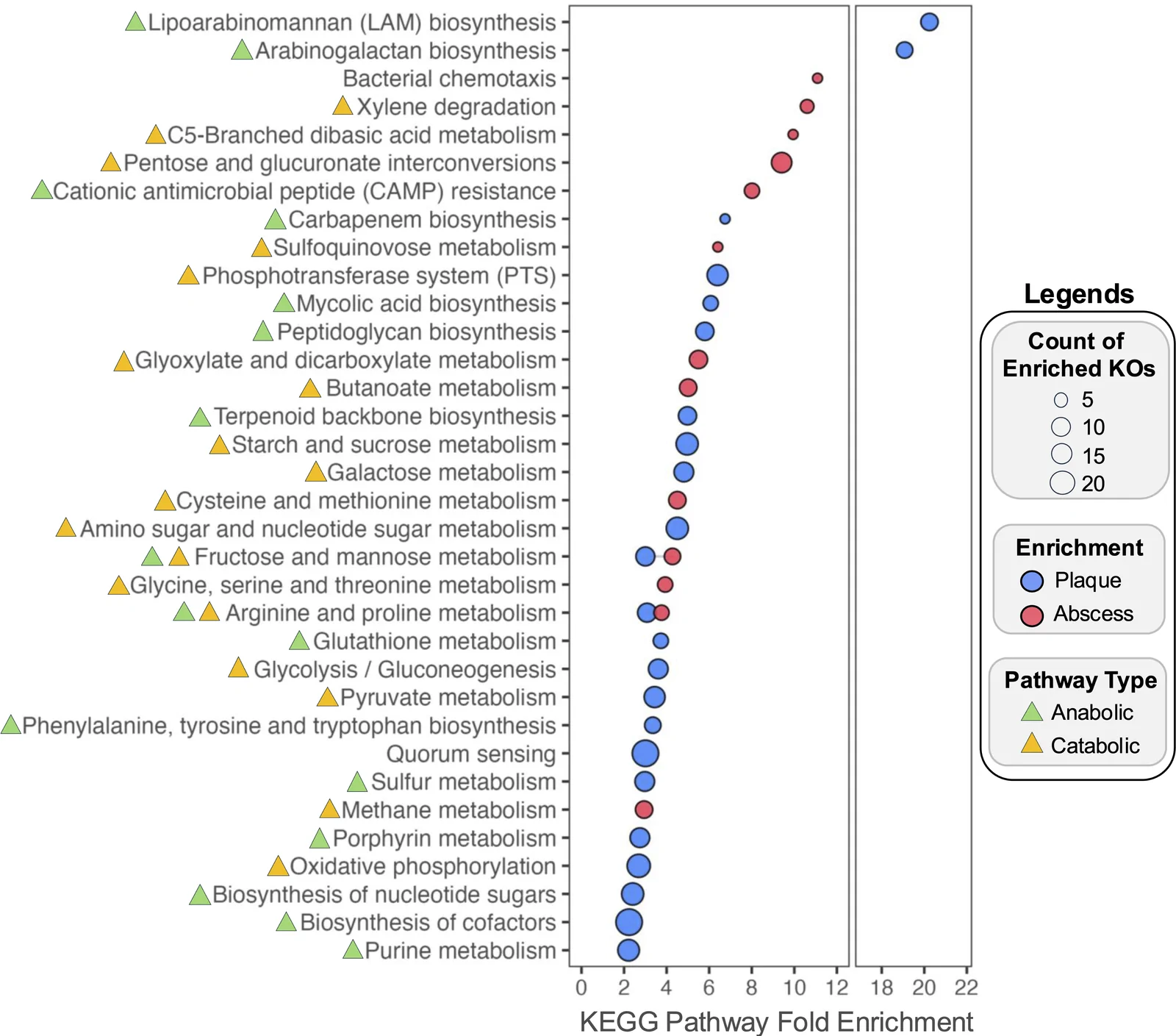
Enrichment of KEGG pathways within plaque and abscess communities. KOs identified by PiCrust2 as differentially enriched in plaque and abscess samples (MaAsLin2 adjusted *q* value < 0.05) were used as inputs to identify the associated KEGG pathways. The *x*-axis displays fold enrichment of the KEGG pathway. Dot size corresponds to the number of unique KOs that were identified as being part of each KEGG pathway (*y*-axis). Triangle color next to each KEGG pathway category indicates whether the pathway is anabolic (green), catabolic (orange), or contains components of both.

Inflammophilic communities are thought to have an obligate dependence upon host responses to provide the nutrients required for proliferation and survival (20, 50). If true, one would predict that such a relationship would correlate with the metabolic pathways enriched in inflammophilic communities. Consequently, we applied two independent algorithmic approaches to investigate inferred metagenomic profiles from the species compositional data. We first employed PICRUSt2 (51) to profile the distribution of enriched functions listed in the KEGG Orthology (KO) database. PICRUSt2 aligns ASVs onto a reference phylogenetic tree and subsequently infers gene and pathway content based upon closely related genomes. MaAslin2 was used to identify specific enrichment of KOs in the plaque and abscess specimens (Table S6). The enriched KOs were next analyzed for their contributions to larger KEGG pathways. This analysis yielded 23 predicted KEGG pathways enriched in dental plaque communities compared to only 13 in the abscesses, suggesting a model with greater metabolic heterogeneity within dental plaque communities (Fig. 8; Table S7). In further support of this notion, the genes identified by PICRUSt2 for the dental plaque-enriched pathways are primarily involved in biosynthetic anabolic metabolism (14 anabolic vs 8 catabolic pathways), whereas the abscess-enriched pathways are extremely biased for catabolism (2 anabolic vs 11 catabolic pathways). Furthermore, the predicted KOs enriched in dental plaque communities largely reflect the Gram-positive species bias in these specimens (Table S7). For example, genes involved in peptidoglycan biosynthesis are enriched as well as the genes used for the biosynthesis of the cell wall lipoglycans of oral *Corynebacterium* species (52–56). Likewise, the genes identified in the quorum sensing category are suggested to be primarily derived from streptococci (Table S7). The results suggest that dental plaque communities exhibit a specific enrichment of biosynthetic anabolic pathways for various amino acids, nucleosides, and cofactors. Energy generation in dental plaque communities is likely heavily biased for glycolysis and oxidative phosphorylation (57) of diverse carbohydrates typically encountered in the diet, including starch, fructose, sucrose, galactose, mannose, and others as well as a concomitant enrichment of the phosphoenolpyruvate:sugar phosphotransferase systems responsible for importing these sugars from the environment (58). Overall, the pathway enrichment results support a model in which the dental plaque communities are largely microaerophilic/facultative and metabolically interdependent, cooperatively synthesizing a large number of the key metabolites required for active growth. In contrast, pathways specifically enriched within abscess communities likely reflect a considerably different ecology and growth strategy. The exceptional bias for catabolic pathway enrichment suggests that abscess communities exhibit a much greater reliance upon the environment to provide the essential metabolites needed for growth. Unlike dental plaque communities, our analysis predicts that abscess communities generate energy principally from anaerobic amino acid fermentation, including cysteine, methionine, glycine, serine, threonine, arginine, and proline as well as phenylalanine, tyrosine, and tryptophan (via the xylene degradation pathway) and glutamate (via the C5-branched dibasic acid pathway; glyoxylate and dicarboxylate pathway) (Table S7). Interestingly, the arginine and proline metabolism KEGG category was also similarly enriched in the dental plaque communities (Fig. 8). However, the dental plaque microbiota genes listed in this category are distinct from those enriched in the abscess communities and are highly likely to be employed for polyamine biosynthesis, rather than ATP generation via arginine and proline catabolism (Table S7). Abscess communities were predicted to exhibit a bias for butanoate metabolism, which is a key ATP-generating pathway used during the fermentation of glutamate, lysine, histidine, cysteine, serine, and methionine (59). Interestingly, the butyrate excreted from butanoate metabolism can directly modulate immune responses and even the epigenetic programming of host cells, potentially influencing host responses to inflammophilic abscess communities (60). Further evidence for probable abscess community dependence upon host-derived proteins can be found among the identified genes categorized in pentose and glucuronate interconversions as well as fructose and mannose metabolism. These genes support the catabolism of unusual sugars commonly present within host protein glycosylations, such as fucose (61) (Fig. 8; Table S7). Conspicuously, abscess communities also exhibited a much greater potential for cationic antimicrobial peptide (CAMP) resistance, which would presumably provide a specific survival advantage when challenged by innate immunity in inflammatory environments (62–64).

As a separate assessment, we repeated the biochemical pathway analysis using a second approach via the Anvi’o pipeline (65). Publicly available genome sequences of representative enriched species (Fig. 5) were downloaded from NCBI RefSeq (Table S8). Functional enrichment profiling of a pangenome constructed using Anvi’o (66, 67) revealed selective enrichment of multiple KOs (*q*-value < 0.05) in both the plaque and abscess communities due to the presence or absence of specific gene clusters in each genome (Table S9). We applied the same KEGG pathway classification analysis to these plaque- and abscess-specific KOs (Table S10). The results largely agree with those of the PICRUSt2 analysis, primarily for the dental plaque communities, whereas far fewer abscess-enriched pathways were detected (Table S10). However, PICRUSt2 and Anvi’o analyses of abscess communities both similarly detected an enrichment of genes related to motility (i.e., chemotaxis and flagellar assembly) as well as glycine degradation (glycine, serine, and threonine metabolism and one carbon pool by folate) (Fig. 8; Table S7 and 10). The Anvi’o analysis identified two pathways that seemingly contradicted the PICRUSt2 results (C5-branched dibasic acid metabolism and terpenoid biosynthesis) (Fig. 8; Table S10). However, none of the genes enriched in either category are shared between the PICRUSt2 and Anvi’o outputs (Table S7 and 10). Furthermore, the abscess-enriched genes listed for C5-branched dibasic acid metabolism primarily function in amino acid degradation, whereas those identified in the plaque communities are related to amino acid biosynthesis (Fig. 8; Table S10). Enrichment of terpenoid biosynthesis genes in plaque communities is primarily attributable to the mevalonate pathway, whereas in the abscess, the identified genes largely function in the non-mevalonate pathway (Table S7 and 10). Thus, no obvious contradictions were detected between the PICRUSt2 and Anvi’o analyses.

## DISCUSSION

In this study, we present a comprehensive ecological analysis of microbiome communities present within a unique cohort of patient-matched clinical specimens of disease-free dental plaque and odontogenic abscess. Although our approach facilitated comparisons of the ecological environments between patient-matched dental plaque and abscess environments, a potential limitation of our sampling strategy is that reference plaque was collected from non-carious primary canines, whereas abscess samples were obtained from primary molars. Non-carious canines were chosen to provide a reliably caries-free plaque reference while avoiding contamination from carious surfaces or from the abscessed tooth itself. Furthermore, primary molars are a reliable source of caries-related extractions in our patient cohort, but few patients also contain caries free contralateral molars suitable for paired analyses. Because plaque and abscesses were sampled from different tooth types, tooth position may have influenced microbial compositional data independent of health status (68, 69). However, our current understanding of site-specificity in the oral cavity does not account for the results detected in our study, especially the predominance of inflammophilic obligate anaerobes in the abscess specimens. It should also be noted that our de-identified specimens did not include patient protected health information, which precluded exclusions for potential confounders like antibiotic use.

Prior studies have demonstrated that human dental plaque is dominated by fast-growing taxa like *Streptococcus* and *Actinomyces* (70–74), whereas odontogenic abscess communities are largely comprised of fastidious, slower growing inflammophilic taxa (21, 75). A unique aspect of the odontogenic abscess is that the dental plaque microbiota that seed the infection become shielded from outside influence following their sequestration within the abscess, whereby they subsequently develop under the exclusive influence of the selective pressures within the inflammatory abscess environment (Fig. 1). This provides an ideal clinical platform to analyze how host inflammatory pressure remodels a eubiotic microbiome community ecology. By leveraging a cohort of patient-matched dental plaque and odontogenic abscess specimens, we could directly observe this remodeling of community ecology while minimizing the confounding influences of inter-individual microbiome variability and specimen contamination during clinical sampling procedures.

Using multiple independent computational techniques, we demonstrated that community composition reliably stratifies plaque from abscess microbiomes. Beyond this result, our analysis highlights a broader methodological issue: the differential abundance method chosen can substantially alter taxa enrichment values, complicating comparison across studies employing disparate approaches (35, 36). Applying three widely used algorithms—MaAsLin2, ALDEx2, and ANCOMBC2—to the same sequence data yielded both overlapping and divergent results. MaAsLin2 returned the highest number of significant taxa across most confidence levels, even under a more stringent filter (*q* < 0.05) than its default (*q* < 0.25); the only exception was low-confidence plaque-enriched species, where ANCOM-BC2 identified more enriched taxa (Fig. 5). The methods also differed in how they handled the matched study design: MaAsLin2 and ALDEx2 yielded clear within-patient differences between plaque and abscess specimens using paired analysis, whereas ANCOMBC2 was markedly more conservative despite the strong overall group separation. This may be due to the relatively small cohort size of the study (Table S11). However, it is worth noting that employing ANCOMBC2 with the less conservative unpaired parameters resulted in fewer significant taxa being reported than either the paired or unpaired output of MaAsLin2 at all levels, highlighting the major differences that can occur in the outputs of the two algorithms. An ideal differential abundance algorithm balances sensitivity and specificity, avoiding both the over-identification of spurious taxa and the under-identification of biologically meaningful ones. These discrepancies observed with our data underscore the importance of combining multiple complementary algorithms (35) to reliably resolve both cohort-level and patient-level differences.

Due to the limited species resolution of 16S rRNA V3V4 gene sequence data for certain bacterial species, some species-level trends may have been poorly captured. We identified one high confidence commensal species in dental plaque (Fig. 5), *Streptococcus sanguinis* (33), and multiple medium-confidence species including *Rothia aeria* (76) and *Corynebacterium durum* (77). In the abscess, the common oral pathobiont *Prevotella nigrescens* was enriched with medium confidence (41) as well as the unnamed oral taxon *Oribacterium* HMT 078. The *Oribacterium* genus is poorly characterized overall, but a variety of *Oribacterium* species are frequently detected in human microbiome studies, often associating with mucosal inflammation (78–85). While little is known about *Oribacterium* HMT 078, it is worth noting that this organism was recently reported to be highly enriched in severe early childhood caries lesions together with *Lacticaseibacillus casei* (86). Severe caries lesions typically precede most endodontic tooth abscesses (Fig. 1), suggesting a potential explanation for the observed enrichment of *Oribacterium* HMT 078 in the abscess specimens. For example, the *Lactobacillus* and *Lacticaseibacillus* genera exhibited weak but detectable enrichment within odontogenic abscesses (Fig. 3C; Fig. 4B), even though neither genus is known to contain oral inflammophiles. Due to their highly acidogenic/aciduric nature, a variety of *Lactobacillus* and *Lacticaseibacillus* species commonly accumulate to exceptionally high levels within deep caries lesions, placing an abundance of these species in close proximity to developing abscess communities (86, 87) (Fig. 1). We propose that a similar phenomenon may contribute to the observed enrichment of *Oribacterium* HMT 078 within the abscess specimens.

Given the limitations of differential abundance analyses with complex microbiome communities, we adapted an LDA topic modeling approach to identify multi-taxa signatures from relative abundance data. A key strength of LDA analysis is that it is far more permissive of the natural variability that exists between human microbiomes (Fig. 7A). This flexibility was especially apparent when applied to genus-level data. For example, the plaque-specific topic 1 was largely driven by *Leptotrichia* and *Capnocytophaga* genera (Fig. S8C), which were similarly found to exhibit high/medium confidence enrichment in the plaque specimens using differential abundance analyses (Fig. 4). However, topic 1 also included *Prevotella* as a major driver despite enrichment of multiple *Prevotella* species in the abscess (Fig. 5). As *Prevotella* is the second-most abundant genus in the oral cavity, it contains a wide variety of both commensal and inflammophilic species (31, 41), which presumably contributed to the genus-level LDA output. We had noted a similar finding with the *Fusobacterium* genus as well (Fig. S8C). Our results suggest that LDA analysis is an underutilized and broadly useful metric for revealing the co-occurring polymicrobial signatures present in clinical specimen cohorts. Importantly, LDA topic modeling of microbiome data captures the strengths of standard differential abundance analysis metrics, while minimizing the negative influence of the inevitable variability that occurs with human clinical specimens.

Similar to our differential abundance analyses, we also employed multiple independent metrics to evaluate the metabolic potential of plaque and abscess communities. Both PICRUSt2 (Table S6) and Anvi’o (Table S9) were used to analyze the distribution of KOs within the oral specimens, and the results of the enrichment analysis were further mapped onto representative KEGG pathways (Table S7 and 10). Both methods have differing limitations (51) but revealed similar trends in community metabolic potential. Dental plaque communities are enriched in biosynthetic anabolic functions used to generate essential metabolites as well as energy-generating catabolic pathways targeting common dietary carbohydrates, implying a fast-growing, highly interdependent microbial ecology supported by interspecies metabolic complementation. In contrast, abscess communities exhibited a conspicuous enrichment of catabolic functions and motility pathways that are likely associated with host tissue destruction, as well as resistance mechanisms targeting host-derived antimicrobials. Thus, our results indicate that inflammophilic community ecology is largely influenced by the selective growth advantage afforded to species that can both resist innate immunity and survive upon the nutrients provided by host inflammatory responses. For example, the specific depletion of highly abundant oral commensal species at sites of inflammation presumably relieves antagonistic growth competition with many pathobiont species. However, it may also force an obligate dependence upon inflammation and/or immunopathology to provide the critical metabolites that would otherwise have been synthesized by cohabitating commensals. Such a result might explain the lack of enriched biosynthetic pathways in the abscess communities. It is conceivable that similar ecological principles to those described in the current study may also define the emergence of the dysbiotic microbiome communities observed at other sites of inflammatory dysbiosis throughout the body. Further insights into the characteristic metabolic capacities supporting inflammophilic communities may ultimately reveal new ecologically focused therapeutic strategies to reestablish eubiosis by depriving access to essential metabolites uniquely supplied by the host.

## MATERIALS AND METHODS

### Sample collection

As part of standard clinical care for one or more abscessed teeth, deidentified clinical specimens were collected from pediatric dental patients, ages 2–12, at Oregon Health & Science University (OHSU) dental clinics. Prior to the start of the project, all protocols were reviewed by the OHSU IRB and classified as “Not Human Subjects Research.” Protected health information was not included with the deidentified specimens. Supragingival plaque was collected using a sterile swab from the facial surface of the non-carious contralateral canine, while abscess samples were collected from the furcation of the extracted, abscessed primary molar. Despite being an anterior tooth, the proximity of the pediatric canine to the primary molars in the pediatric arch was anticipated to reduce the ecological differences reported for the anterior-posterior gradient of adult oral microbiota. In the event of a draining abscess, purulence was sampled from the site of the fistula. Samples were transferred to the anaerobic chamber, mixed with glycerol freezing media, and stored at −80°C.

### DNA collection and sequencing preparation

DNA was isolated from samples stored in DNA/RNA shield using the ZymoBIOMICS DNA Miniprep kit (Zymo Research) per the manufacture’s recommendation, using a double-elution protocol with 50 µL of H2O warmed to 60°C and no Zymo-Spin III-HRC filtering step. 16S rRNA gene sequencing was performed as previously reported (88, 89). Briefly, the V3‐V4 variable region of the 16S rRNA gene was amplified using gene‐specific primers with Illumina adapter overhang sequences (5′‐TCGTCGGCAGCGTCAGATGTGTATAAGAGACAGCCTACGGGNGGCWGCAG‐3′ and 5′‐GTCTCGTGGGCTCGGAGATGTGTATAAGAGACAGGACTACHVGGGTATCTAATCC‐3′) using 12.5 µL Kapa HiFi Hot Start ReadyMix, 2.5 µL gDNA, and 10 µL sterile H_2_O or with the following protocol: 95°C for 3 min; either 35 or 40 cycles at 95°C for 30 s, 55°C for 30 s, 72°C for 30 s; 72°C for 5 min using 40 cycles of amplification due to low bacterial abundance in the original sample. Amplicons were cleaned using AMPure XP beads and eluted into 50 µL of H_2_O. Indices were attached using 8 cycles of the same PCR protocol, and amplicons were cleaned with AMPure XP beads. The size of select products was verified using gel electrophoresis. Twenty-five microliters of each sample was submitted to the McLean Lab at the University of Washington for Illumina MiSeq sequencing (2 × 300 bp, 10% PhiX spike).

### Community analysis and bioinformatics

QIIME2 (version 2022.2.0) (90) and DADA2 (91) within the QIIME2 package were used to trim and classify sequences using the feature-classifier classify-consensus-blast referencing the Human Oral Microbiome Database (version 15.23). R studio Phyloseq (version 1.52.0) (92) was used for subsequent analysis. Contaminates listed in Table S12 were removed from the analysis, and ASVs found only in the PCR and reagent blank samples were removed. Additionally, only samples where both plaque and abscess samples contained >1,000 reads were retained. The ggplot2 and pheatmap packages in R were used to produce figures.

### Differential abundance analysis

Three differential abundance algorithms were tested: Microbiome Multivariable Associations with Linear Models (MaAsLin2, version 1.22.0) (37), Analysis of Compositions of Microbiomes with Bias Correction (ANCOMBC2, version 2.10.2) (38), and Analysis Of Differential Abundance Taking Sample and Scale Variation Into Account (ALDEx2, run through the microbiomeMarker package version 1.13.2) (39). Model parameters for MaAsLin2 and ALDEx2 were chosen through parameter sweeps, which considered the number of differential taxa found through each combination of data transformation, normalization, and method. All three methods offer options to account for the paired nature of our data set, and each was run both with and without patient pairing at the phylum, genus, and species levels. MaAsLin2 was run with a linear model, CSS normalization, and log transformation of count data, with patient included as a random effect for the paired analysis and a *q*-value significance threshold of 0.05. ALDEx2 was run using a log10 transformation, CSS normalization of count data, and a Wilcoxon signed-rank test for significance (Benjamini–Hochberg-adjusted *P* < 0.05), with sample-pairing specified. ANCOMBC2 used a Holm *p*-value adjustment and a 0.05 significance threshold, but due to the extremely sparse results given when including sample pairing (Table S11), ANCOMBC2 was run without a sample-pairing parameter.

### Ordination

To choose the ordination method and parameters, we used an iterative parameter sweep to select the most appropriate distance measure and data transformation for each ordination method (Principal Coordinates Analysis, Principal Component Analysis, or Non-metric multidimensional scaling). This parameter sweep found the combination of distance measures (Jaccard, Bray-Curtis, or Euclidean) and data transformations (total sum scaling and centered log ratio) that minimized the correlation (Spearman’s rho) with library size for the first axis of variation, maximized the silhouette score within each type (plaque and abscess), and considered the percent variation captured by the first two axes. Considering these parameters, we chose PCoA of relative abundance values using Bray-Curtis distance as our final ordination model. Community separation by sample type was assessed with silhouette scores and PERMANOVA (vegan version 2.7-5 on Bray-Curtis distances with permutations restricted within patient pairs), with betadisper/permutest used to test whether group differences reflected unequal multivariate dispersion rather than a true compositional shift.

### Random forest

Relative abundance taxa values were used as input to the random forest model agglomerated at the genus level. The caret package in R (version 7.0-1) was used to construct a random forest classifier to identify taxa most informative for plaque vs abscess classification, with variable importance quantified by mean decrease in Gini impurity and assessed across stratified fourfold cross-validation.

### Metabolic profiling

PICRUSt2 (51) (version 2.6.2) was run from a conda environment using trimmed representative sequences output by the Qiime2 pipeline. The results of the KO analysis were analyzed for differential abundance using MaAsLin2 (CLR normalization, no transformation). Lists of significant results (*q* value < 0.05) for KOs differentially enriched in plaque and abscess samples (Table S6) were used for input into KEGG enrichment analysis using the ClusterProfiler enrichKEGG command. Results for both the plaque and abscess specific analysis were combined and shown in Fig. 8, with the fold enrichment of these KEGG pathways on the *x*-axis.

An Anvi’o (65) (version 8) pangenome was constructed from all the species identified as being differentially abundant in either plaque or abscess communities using any of the three methods (Table S4). RefSeq representative genomes for each species were collected from NCBI (Table S8). Taxa without any available genomes were omitted from the pangenome analysis. Anvi’o was used to construct the pangenome, and functional enrichment in either plaque or abscess communities was computed for KOs using the anvi-compute-functional-enrichment-in-pan (67) command. Functional enrichment results were filtered by adjusted *q*-value (*q* < 0.05) (Table S10). As with the Picrust2 results, KOs that were differentially enriched in plaque and abscess samples were used as input into the enrichKEGG function from ClusterProfiler, and the resulting enrichments are shown in Fig. S10.

### Topic modeling

For taxa, relative abundance values agglomerated at the genus level were used as input for topic modeling. A scaling factor of 1,000 was chosen based on silhouette score optimization for both plaque and abscess communities. The number of topics was determined from a set of four different metrics; as these metrics have not been explicitly named by the authors, we refer to these four different approaches by the first author of the study: CaoJuan2009 (93), Arun2010 (94), Deveaud2014 (95), and Griffiths2004 (96). CaoJuan2009, Arun2010, and Deveaud2014 approaches aim to either minimize the similarity or the difference between the number of topics, essentially all operating under the same intuition that non-redundant and dissimilar topics lead to the best K. Griffiths2004 approach uses a standard Bayesian Model selection strategy that selects the number of topics that maximizes likelihood. The LDA function from the topicmodels package (version 0.2-17) was used to build an LDA models for species and genus level data. A Wilcoxon Signed-rank test with FDR *P*-value correction was used to assess the differential significance of topic membership by type.

## Data Availability

All code is publicly available on github (https://github.com/kriegerm/PA_cohort_analysis). 16S rRNA V3V4 gene sequencing reads have been deposited on Sequence Read Archive in PRJNA1312071.

## SUPPLEMENTAL MATERIAL

Supplemental figures spectrum.01550-26-s0001.pdf Fig. S1 to S5.

Supplemental figures spectrum.01550-26-s0002.pdf Fig. S6 to S10.

Supplemental legends spectrum.01550-26-s0003.docx Legends Fig. S1 to S10 and Tables S1 to S12.

Supplemental tables spectrum.01550-26-s0004.xlsx Tables S1 to S12.

ASM does not own the copyrights to Supplemental Material that may be linked to, or accessed through, an article. The authors have granted ASM a non-exclusive, world-wide license to publish the Supplemental Material files. Please contact the corresponding author directly for reuse.
