# Supplementary material for: Paired oral clinical specimens reveal the underlying ecology supporting the emergence of inflammophilic microbiome communities
Source: Microbiol Spectr. 2026 Sep 14;14(10):e01550-26. doi: 10.1128/spectrum.01550-26 (PMC13689325; doi:10.1128/spectrum.01550-26)
Supplement: Supplementary file 1 [file spectrum.01550-26-s0001.pdf]

A

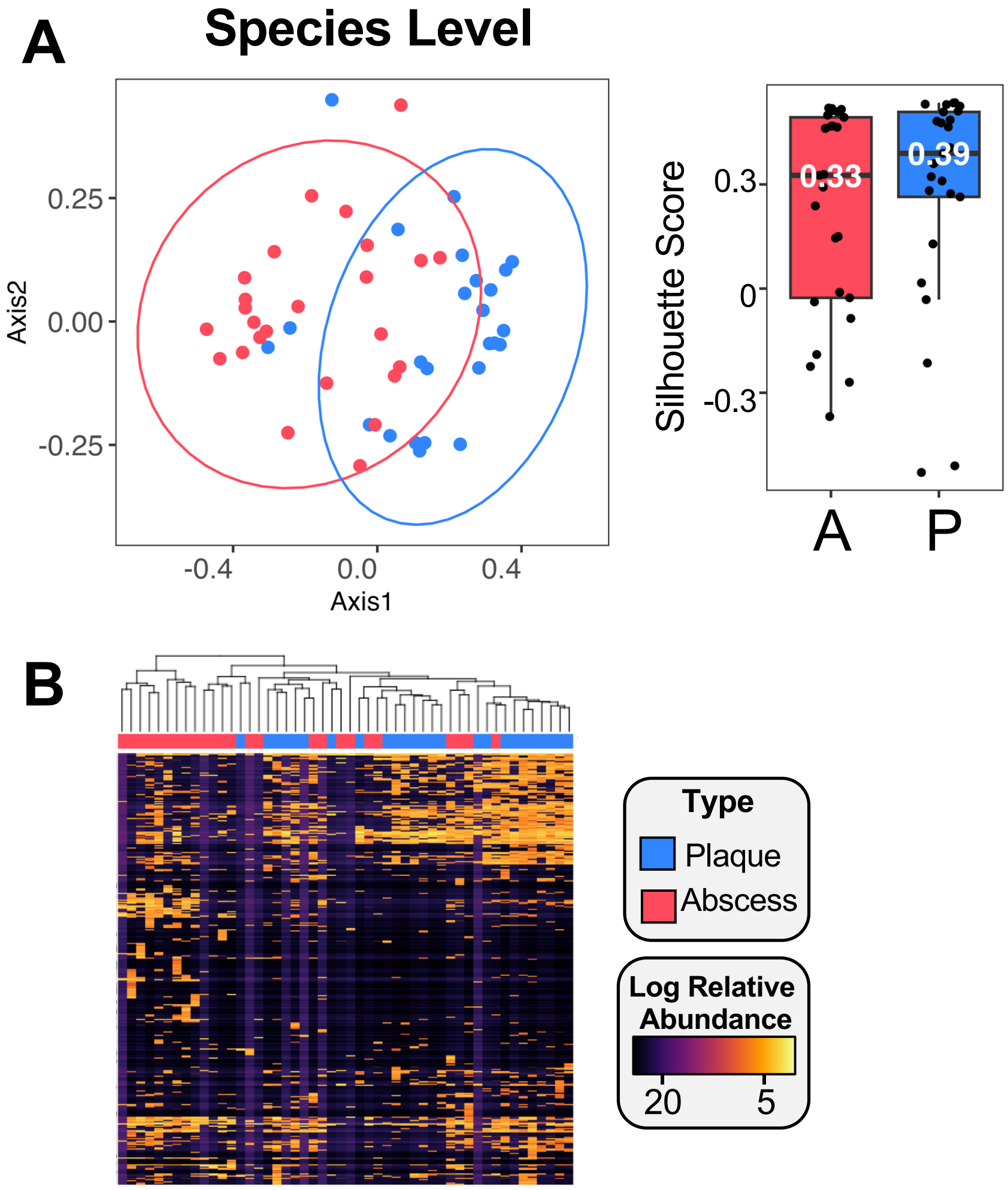

Species Level
Axis2
0.25
0.00
-0.25
-0.4
0.0
0.4
Axis1
Silhouette Score
0.3
0
-0.3
0.33
0.39
A
P
B
Type
Plaque
Abscess
Log Relative Abundance
20
5

Figure S1

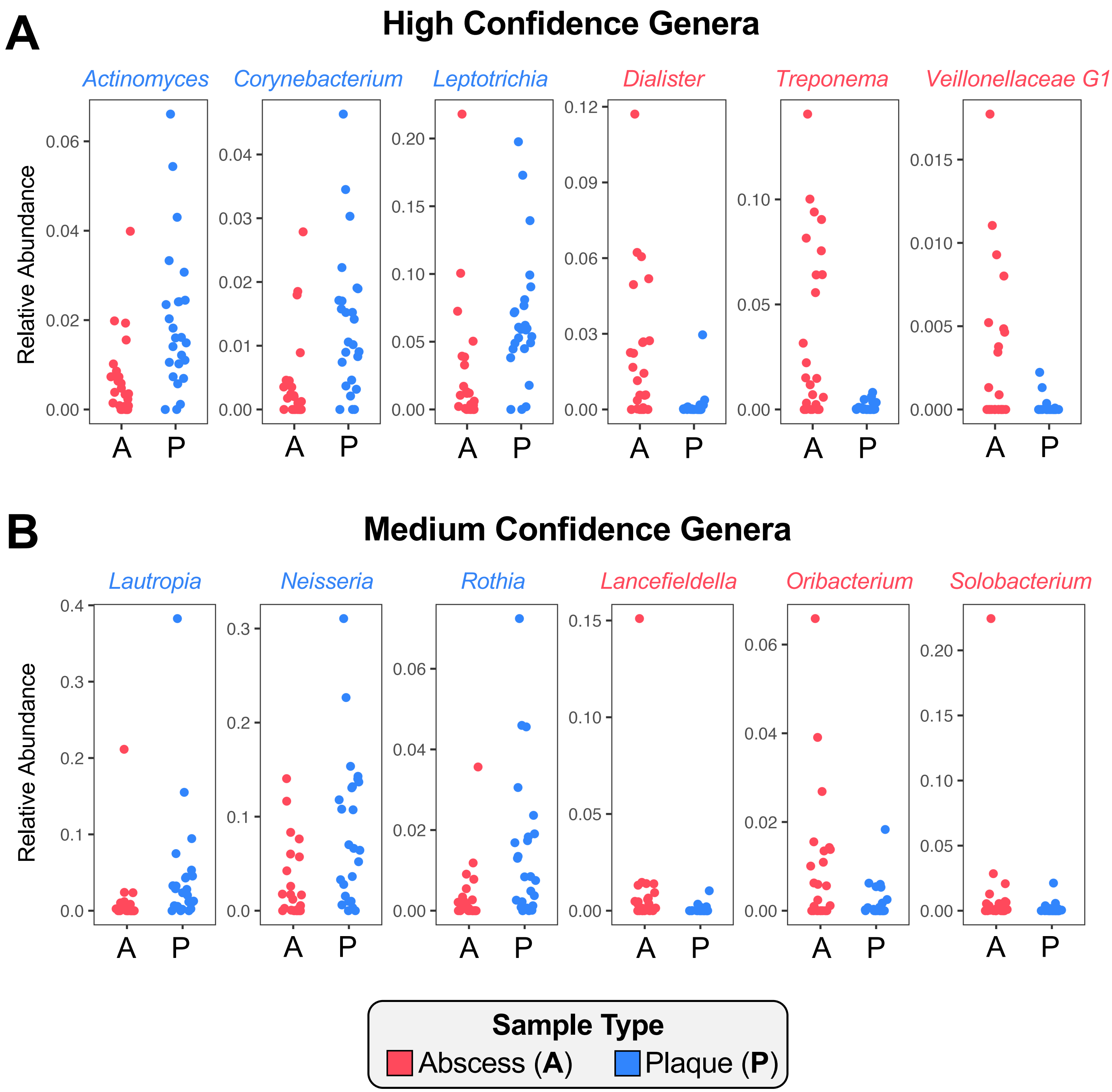
A
High Confidence Genera
Actinomyces
Corynebacterium
Leptotrichia
Dialister
Treponema
Veillonellaceae G1
Relative Abundance
A
P
B
Medium Confidence Genera
Lautropia
Neisseria
Rothia
Lancefieldella
Oribacterium
Solobacterium
Relative Abundance
A
P
Sample Type
Abscess (A)
Plaque (P)

Figure S2

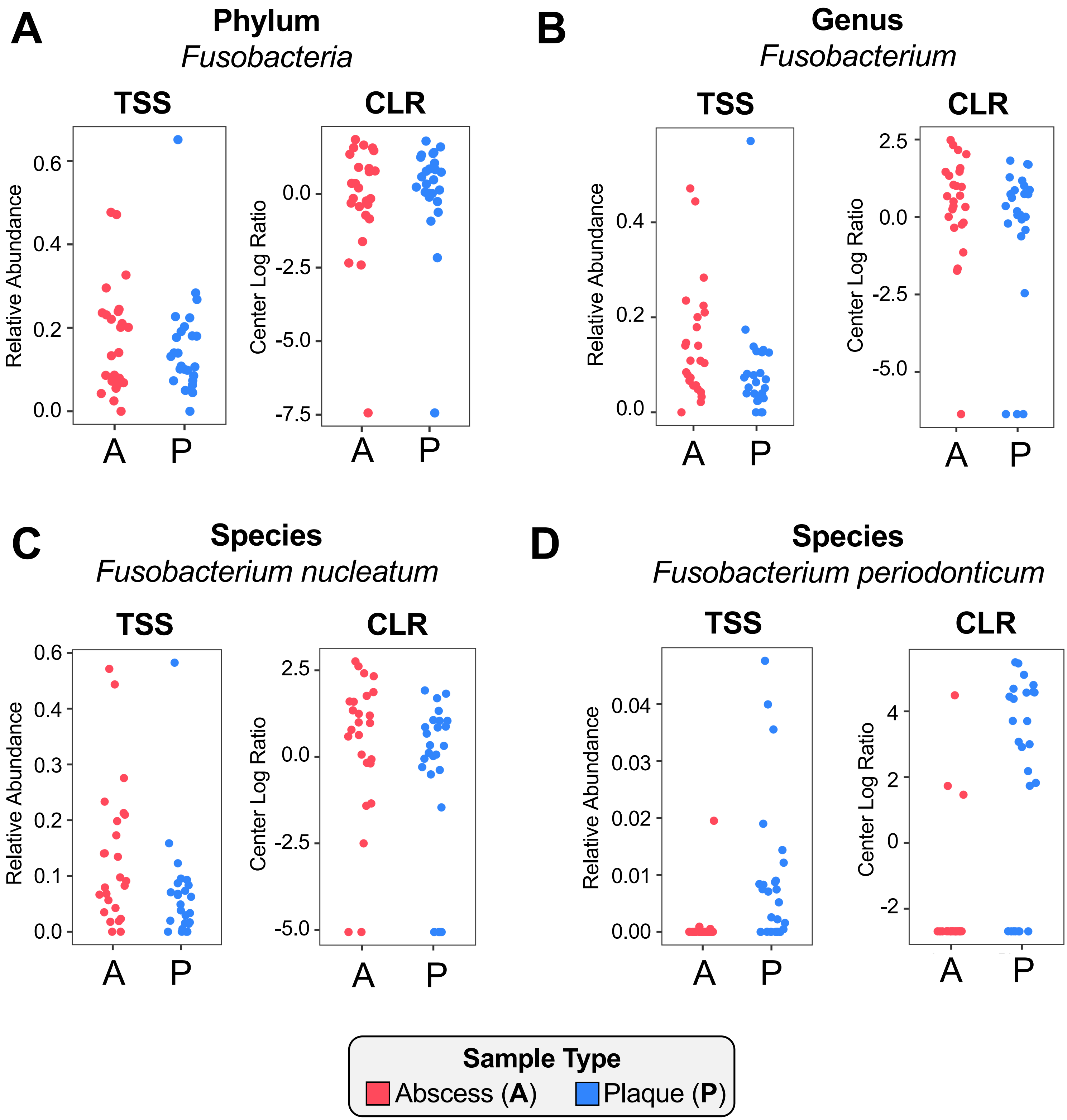
A
Phylum
Fusobacteria
TSS
CLR
Relative Abundance
Center Log Ratio
0.0
0.2
0.4
0.6
0.0
-2.5
-5.0
-7.5
A
P
B
Genus
Fusobacterium
TSS
CLR
Relative Abundance
Center Log Ratio
0.0
0.2
0.4
2.5
0.0
-2.5
-5.0
A
P
C
Species
Fusobacterium nucleatum
TSS
CLR
Relative Abundance
Center Log Ratio
0.0
0.1
0.2
0.3
0.4
0.6
2.5
0.0
-2.5
-5.0
A
P
D
Species
Fusobacterium periodonticum
TSS
CLR
Relative Abundance
Center Log Ratio
0.00
0.01
0.02
0.03
0.04
4
2
0
-2
A
P
Sample Type
Abscess (A)
Plaque (P)

Figure S3

A

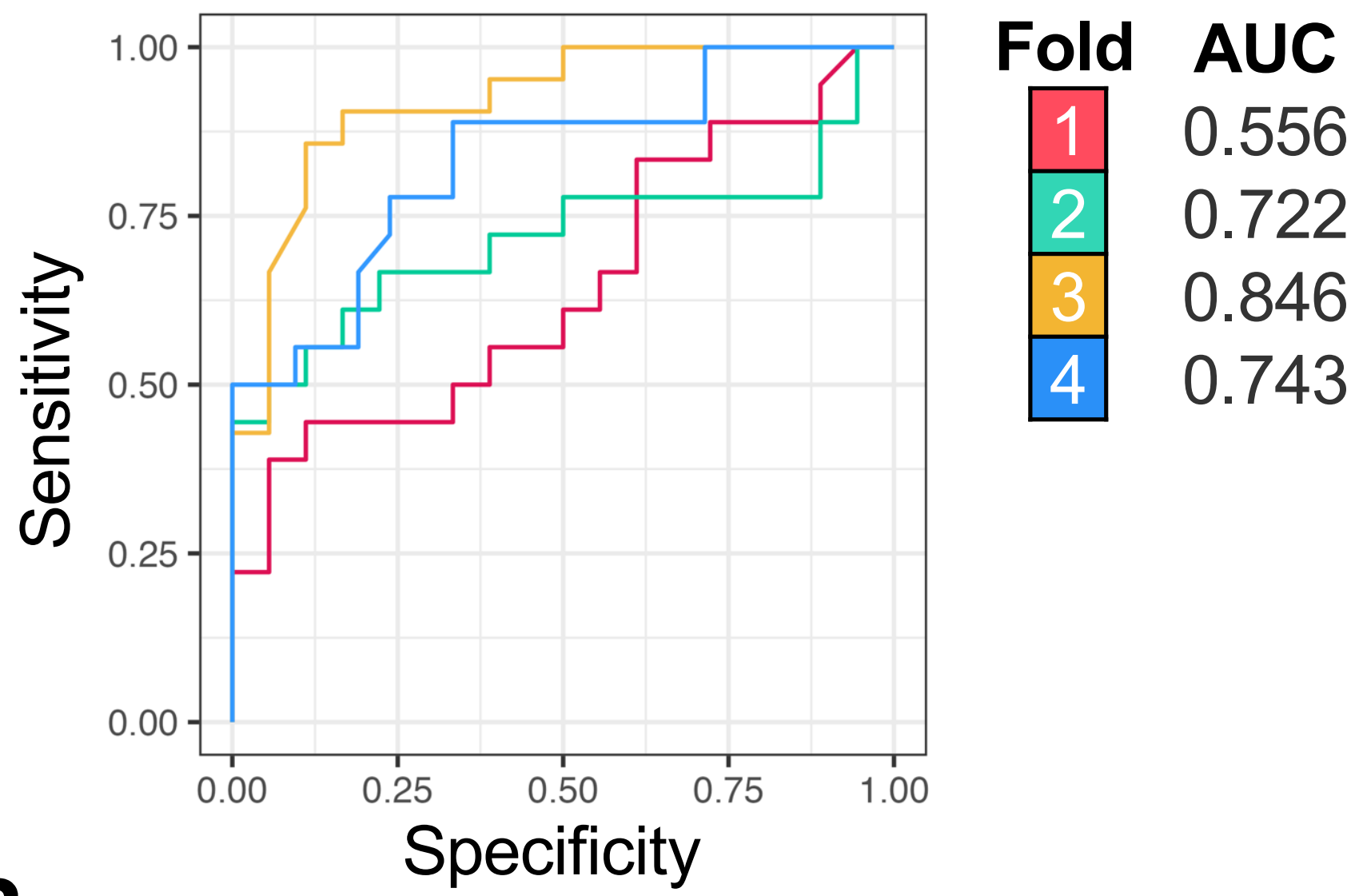

B

Feature Rank per Fold

1	2	3	4	Fold
1	1	10	2	*Streptococcus sanguinis*
16	5	8	14	*Dialister invisus*
2	3	1	42	*Alloprevotella tannerae*
9	62	4	1	*Fusobacterium periodonticum*
10	14	18	75	*Neisseria elongata*
24	60	36	6	*Actinomyces oris*
12	7	55	61	*Porphyromonas endodontalis*
89	13	40	5	*Lautropia mirabilis*
79	37	46	3	*Leptotrichia sp. HMT 215*
13	9	26	123	*Prevotella sp. HMT 292*
27	12	6	134	*Rothia aeria*
35	4	12	137	*Prevotella oris*
48	8	115	60	*Neisseria flava*
3	89	141	25	*Veillonellaceae G-1 bacterium HMT 150*
5	25	2	315	*Oribacterium sp. HMT 078*
33	22	297	7	*Porphyromonas pasteri*
319	32	3	9	*Leptotrichia hofstadii*
321	2	77	10	*Capnocytophaga sputigena*
34	21	363	4	*Kingella oralis*
6	410	69	13	*Actinomyces massiliensis*
433	54	7	12	*Corynebacterium matruchotii*
172	30	9	296	*Lancefieldella rimae*
4	181	5	386	*Treponema sp. HMT 231*
7	86	384	153	*Abiotrophia defectiva*
86	174	385	8	*Arachnia propionica*
8	390	80	303	*Peptococcus sp. HMT 167*
193	6	431	397	*Neisseria oralis*
346	10	440	291	*Neisseria cinerea*

Feature Rank
- Most important
- Least important

Figure S4

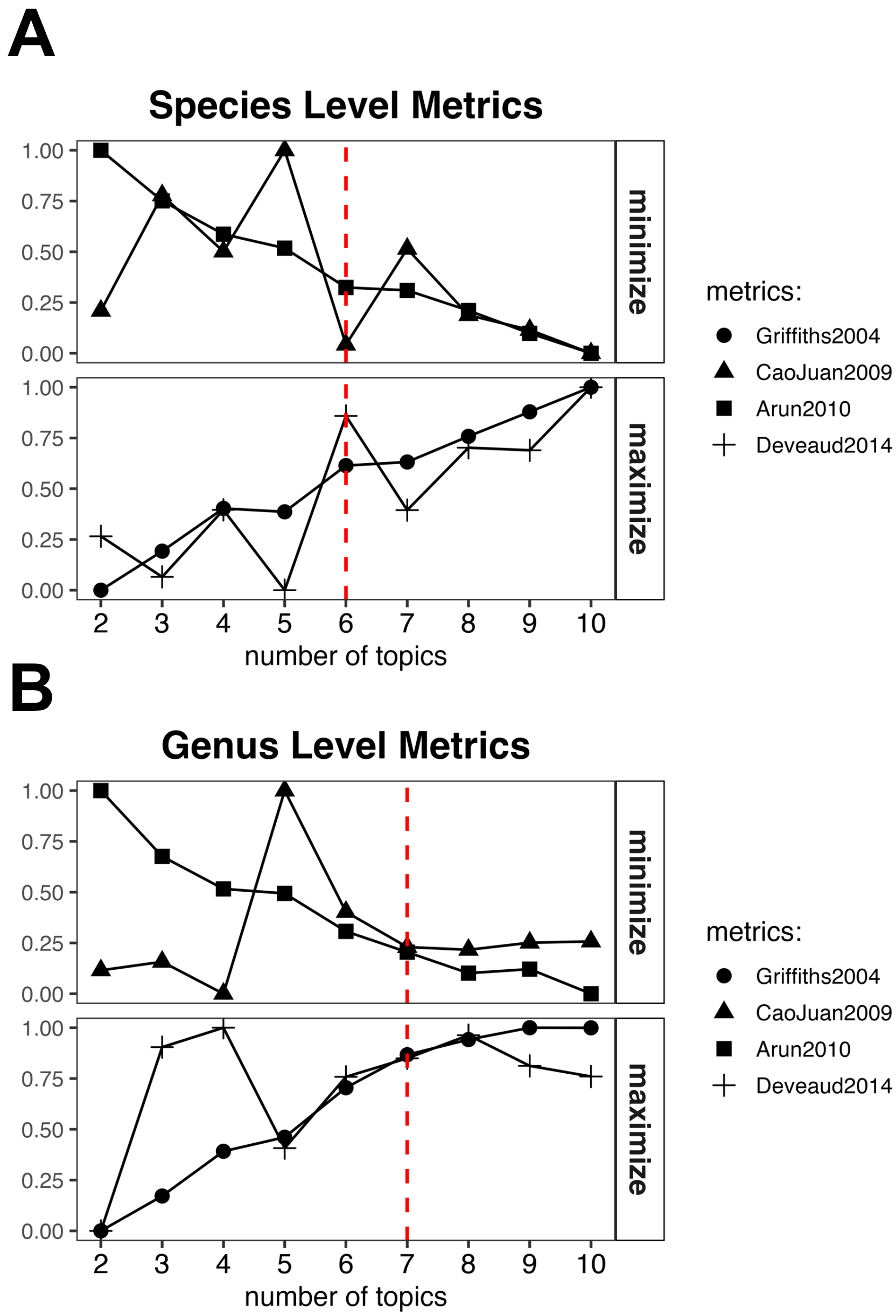
A
Species Level Metrics
minimize
maximize
1.00
0.75
0.50
0.25
0.00
2
3
4
5
6
7
8
9
10
number of topics
metrics:
Griffiths2004
CaoJuan2009
Arun2010
Deveaud2014
B
Genus Level Metrics
minimize
maximize
number of topics
metrics:
Griffiths2004
CaoJuan2009
Arun2010
Deveaud2014

Figure S5
