# Supplementary material for: Paired oral clinical specimens reveal the underlying ecology supporting the emergence of inflammophilic microbiome communities
Source: Microbiol Spectr. 2026 Sep 14;14(10):e01550-26. doi: 10.1128/spectrum.01550-26 (PMC13689325; doi:10.1128/spectrum.01550-26)
Supplement: Supplementary file 2 [file spectrum.01550-26-s0002.pdf]

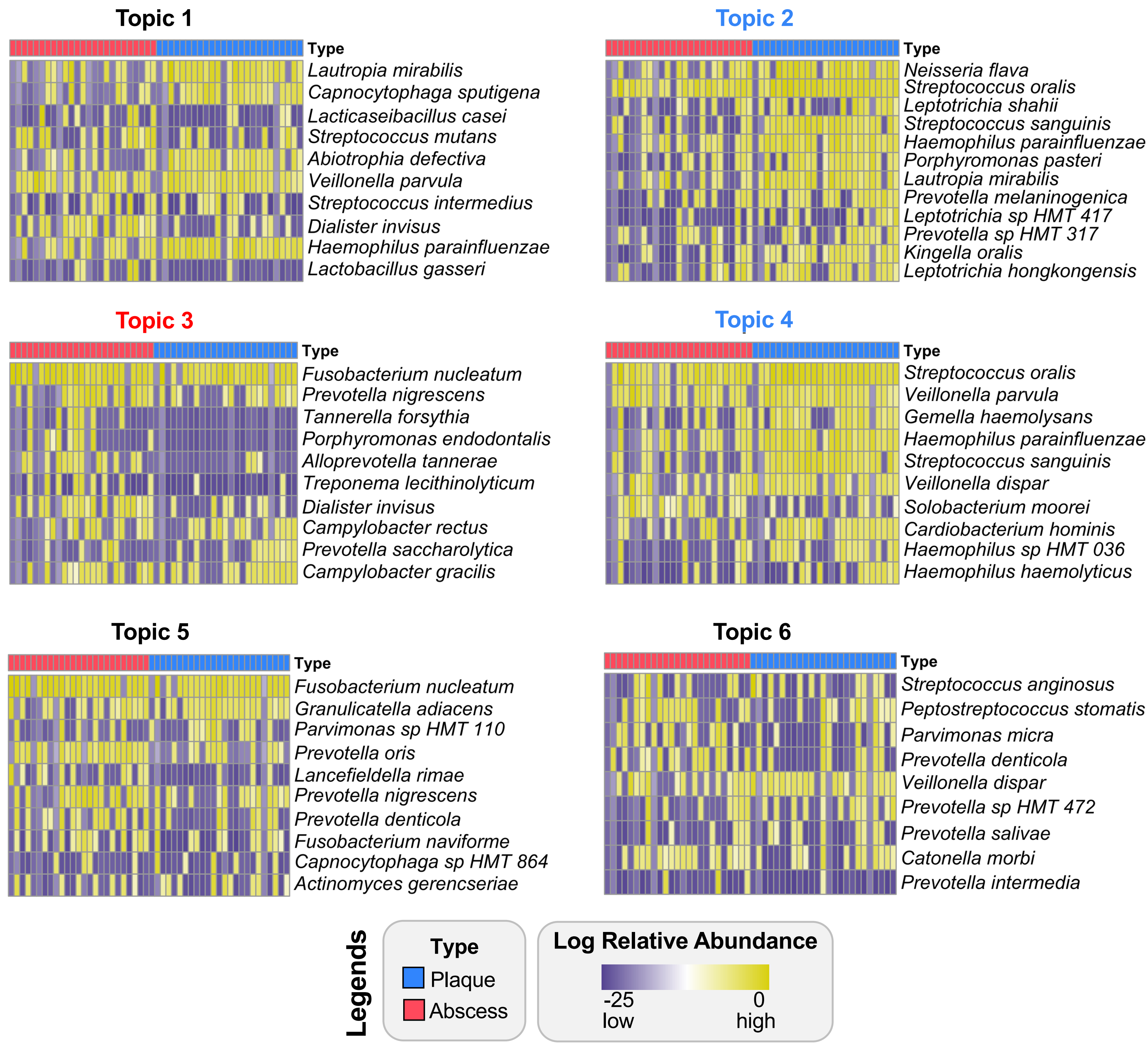

Topic 1
Type
Lautropia mirabilis
Capnocytophaga sputigena
Lacticaseibacillus casei
Streptococcus mutans
Abiotrophia defectiva
Veillonella parvula
Streptococcus intermedius
Dialister invisus
Haemophilus parainfluenzae
Lactobacillus gasseri
Topic 2
Type
Neisseria flava
Streptococcus oralis
Leptotrichia shahii
Streptococcus sanguinis
Haemophilus parainfluenzae
Porphyromonas pasteri
Lautropia mirabilis
Prevotella melaninogenica
Leptotrichia sp HMT 417
Prevotella sp HMT 317
Kingella oralis
Leptotrichia hongkongensis
Topic 3
Type
Fusobacterium nucleatum
Prevotella nigrescens
Tannerella forsythia
Porphyromonas endodontalis
Alloprevotella tannerae
Treponema lecithinolyticum
Dialister invisus
Campylobacter rectus
Prevotella saccharolytica
Campylobacter gracilis
Topic 4
Type
Streptococcus oralis
Veillonella parvula
Gemella haemolysans
Haemophilus parainfluenzae
Streptococcus sanguinis
Veillonella dispar
Solobacterium moorei
Cardiobacterium hominis
Haemophilus sp HMT 036
Haemophilus haemolyticus
Topic 5
Type
Fusobacterium nucleatum
Granulicatella adiacens
Parvimonas sp HMT 110
Prevotella oris
Lancefieldella rimae
Prevotella nigrescens
Prevotella denticola
Fusobacterium naviforme
Capnocytophaga sp HMT 864
Actinomyces gerencseriae
Topic 6
Type
Streptococcus anginosus
Peptostreptococcus stomatis
Parvimonas micra
Prevotella denticola
Veillonella dispar
Prevotella sp HMT 472
Prevotella salivae
Catonella morbi
Prevotella intermedia
Legends
Type
Plaque
Abscess
Log Relative Abundance
-25
low
0
high

Figure S6

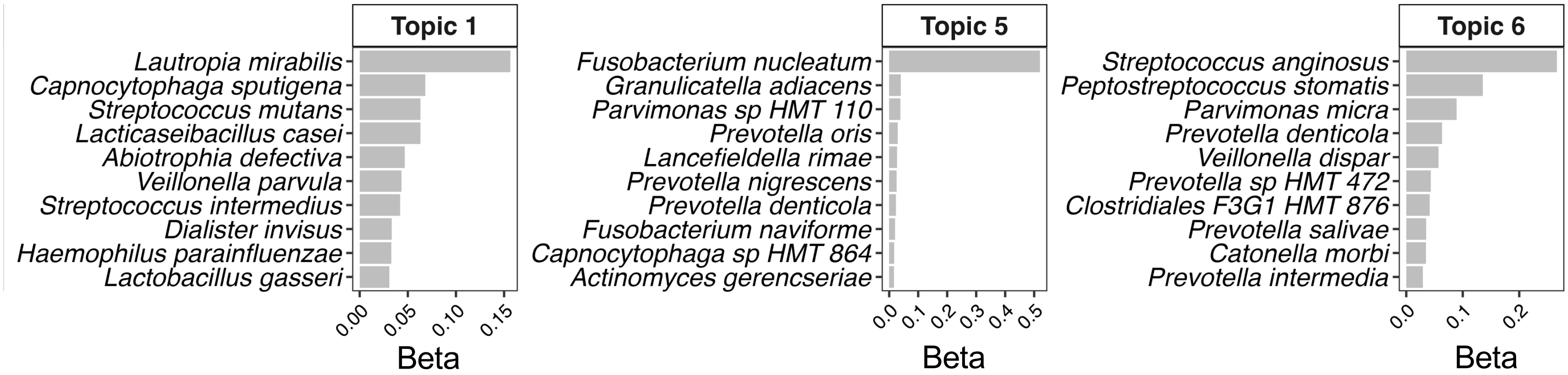

Topic 1
Lautropia mirabilis
Capnocytophaga sputigena
Streptococcus mutans
Lacticaseibacillus casei
Abiotrophia defectiva
Veillonella parvula
Streptococcus intermedius
Dialister invisus
Haemophilus parainfluenzae
Lactobacillus gasseri
0.00
0.05
0.10
0.15
Beta
Topic 5
Fusobacterium nucleatum
Granulicatella adiacens
Parvimonas sp HMT 110
Prevotella oris
Lancefieldella rimae
Prevotella nigrescens
Prevotella denticola
Fusobacterium naviforme
Capnocytophaga sp HMT 864
Actinomyces gerencseriae
0.0
0.1
0.2
0.3
0.4
0.5
Beta
Topic 6
Streptococcus anginosus
Peptostreptococcus stomatis
Parvimonas micra
Prevotella denticola
Veillonella dispar
Prevotella sp HMT 472
Clostridiales F3G1 HMT 876
Prevotella salivae
Catonella morbi
Prevotella intermedia
0.0
0.1
0.2
Beta

Figure S7

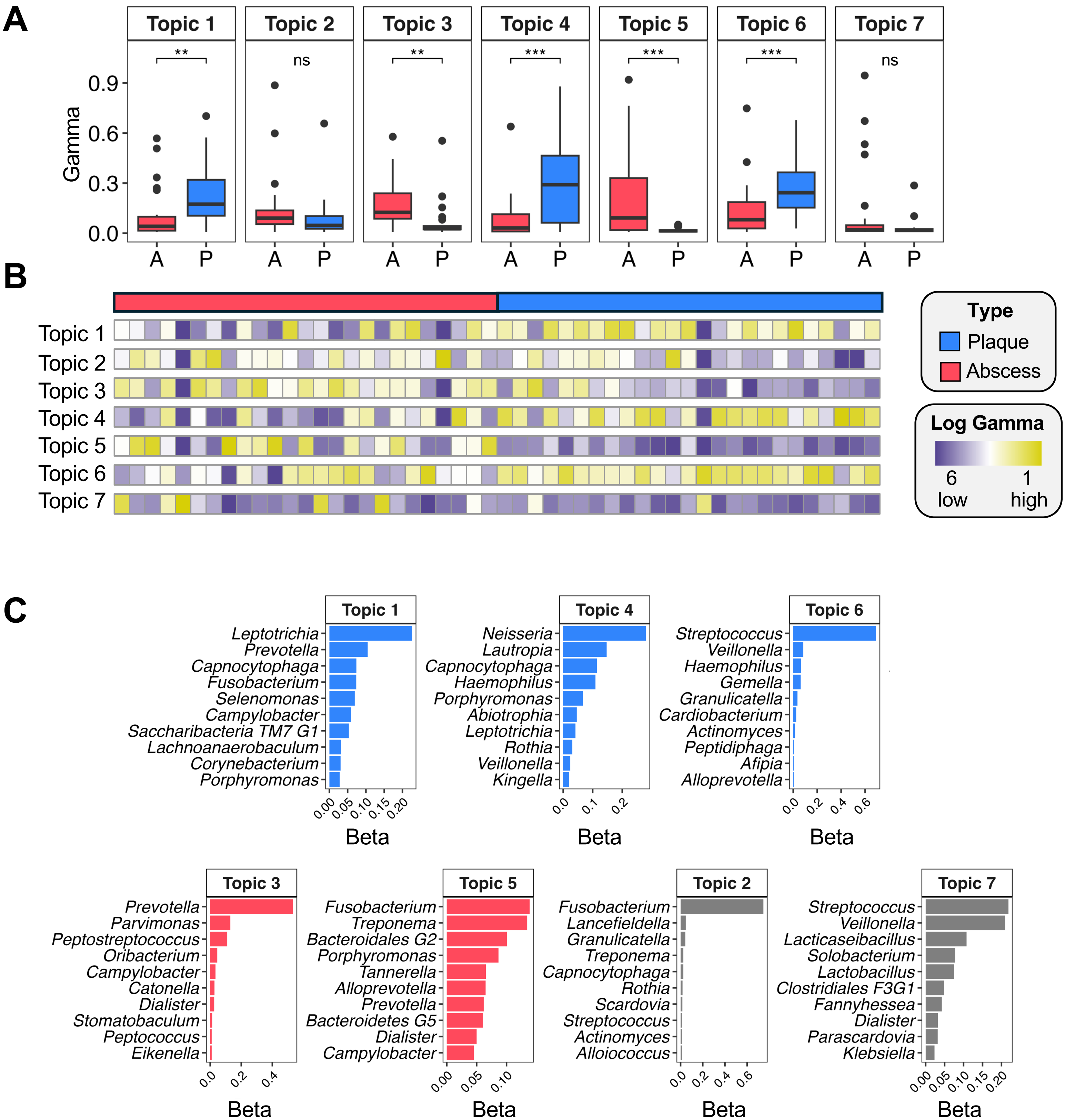
A
Topic 1
Topic 2
Topic 3
Topic 4
Topic 5
Topic 6
Topic 7
Gamma
0.0
0.3
0.6
0.9
**
ns
**
***
***
***
ns
A
P
B
Topic 1
Topic 2
Topic 3
Topic 4
Topic 5
Topic 6
Topic 7
Type
Plaque
Abscess
Log Gamma
6
low
1
high
C
Topic 1
Leptotrichia
Prevotella
Capnocytophaga
Fusobacterium
Selenomonas
Campylobacter
Saccharibacteria TM7 G1
Lachnoanaerobaculum
Corynebacterium
Porphyromonas
Beta
Topic 4
Neisseria
Lautropia
Capnocytophaga
Haemophilus
Porphyromonas
Abiotrophia
Leptotrichia
Rothia
Veillonella
Kingella
Beta
Topic 6
Streptococcus
Veillonella
Haemophilus
Gemella
Granulicatella
Cardiobacterium
Actinomyces
Peptidiphaga
Afipia
Alloprevotella
Beta
Topic 3
Prevotella
Parvimonas
Peptostreptococcus
Oribacterium
Campylobacter
Catonella
Dialister
Stomatobaculum
Peptococcus
Eikenella
Beta
Topic 5
Fusobacterium
Treponema
Bacteroidales G2
Porphyromonas
Tannerella
Alloprevotella
Prevotella
Bacteroidetes G5
Dialister
Campylobacter
Beta
Topic 2
Fusobacterium
Lancefieldella
Granulicatella
Treponema
Capnocytophaga
Rothia
Scardovia
Streptococcus
Actinomyces
Alloiococcus
Beta
Topic 7
Streptococcus
Veillonella
Lacticaseibacillus
Solobacterium
Lactobacillus
Clostridiales F3G1
Fannyhessea
Dialister
Parascardovia
Klebsiella
Beta

Figure S8

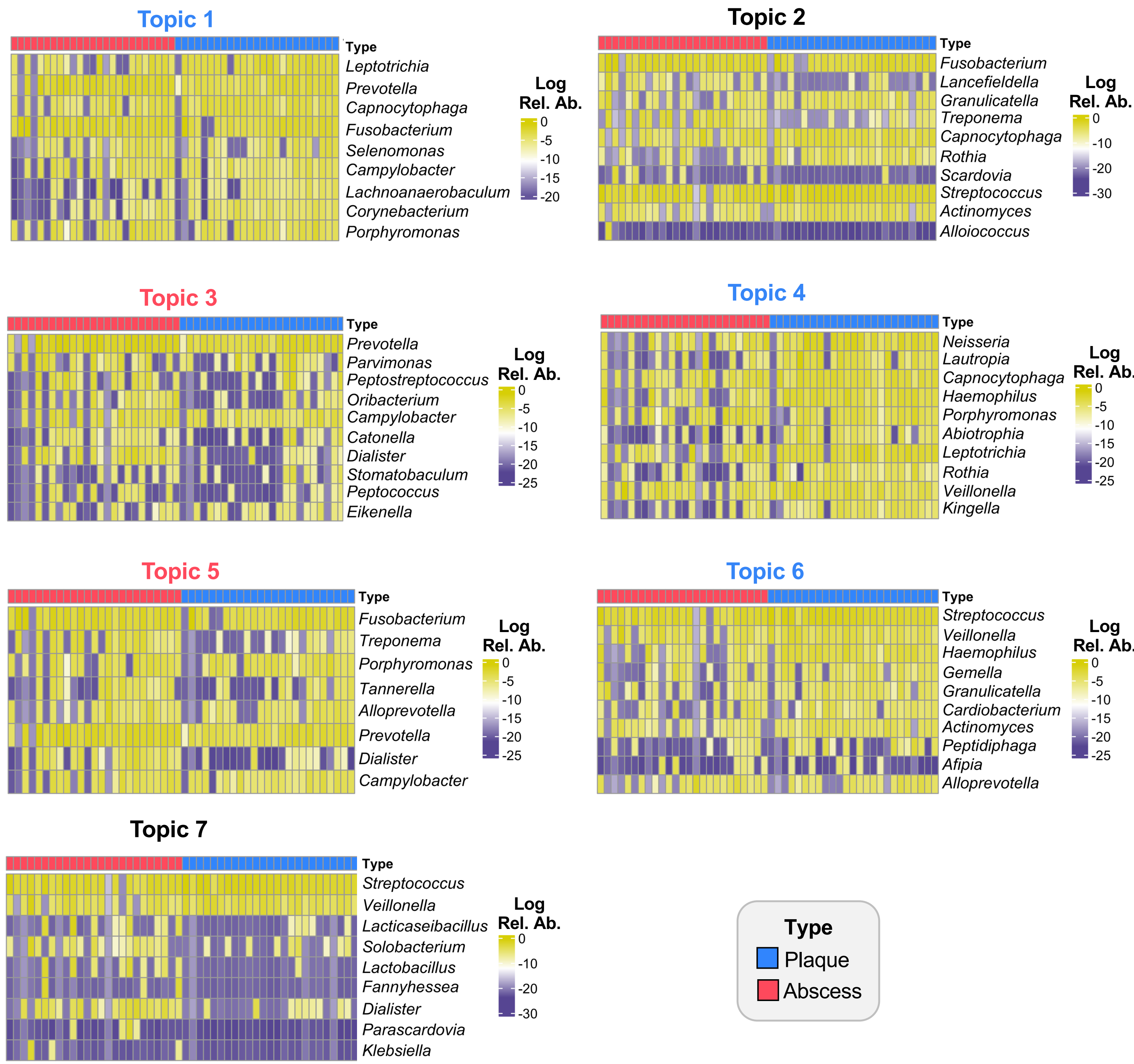

Topic 1
Type
Leptotrichia
Prevotella
Capnocytophaga
Fusobacterium
Selenomonas
Campylobacter
Lachnoanaerobaculum
Corynebacterium
Porphyromonas
Log Rel. Ab.
0
-5
-10
-15
-20
Topic 2
Type
Fusobacterium
Lancefieldella
Granulicatella
Treponema
Capnocytophaga
Rothia
Scardovia
Streptococcus
Actinomyces
Alloiococcus
Log Rel. Ab.
0
-10
-20
-30
Topic 3
Type
Prevotella
Parvimonas
Peptostreptococcus
Oribacterium
Campylobacter
Catonella
Dialister
Stomatobaculum
Peptococcus
Eikenella
Log Rel. Ab.
0
-5
-10
-15
-20
-25
Topic 4
Type
Neisseria
Lautropia
Capnocytophaga
Haemophilus
Porphyromonas
Abiotrophia
Leptotrichia
Rothia
Veillonella
Kingella
Log Rel. Ab.
0
-5
-10
-15
-20
-25
Topic 5
Type
Fusobacterium
Treponema
Porphyromonas
Tannerella
Alloprevotella
Prevotella
Dialister
Campylobacter
Log Rel. Ab.
0
-5
-10
-15
-20
-25
Topic 6
Type
Streptococcus
Veillonella
Haemophilus
Gemella
Granulicatella
Cardiobacterium
Actinomyces
Peptidiphaga
Afipia
Alloprevotella
Log Rel. Ab.
0
-5
-10
-15
-20
-25
Topic 7
Type
Streptococcus
Veillonella
Lacticaseibacillus
Solobacterium
Lactobacillus
Fannyhessea
Dialister
Parascardovia
Klebsiella
Log Rel. Ab.
0
-10
-20
-30
Type
Plaque
Abscess

Figure S9

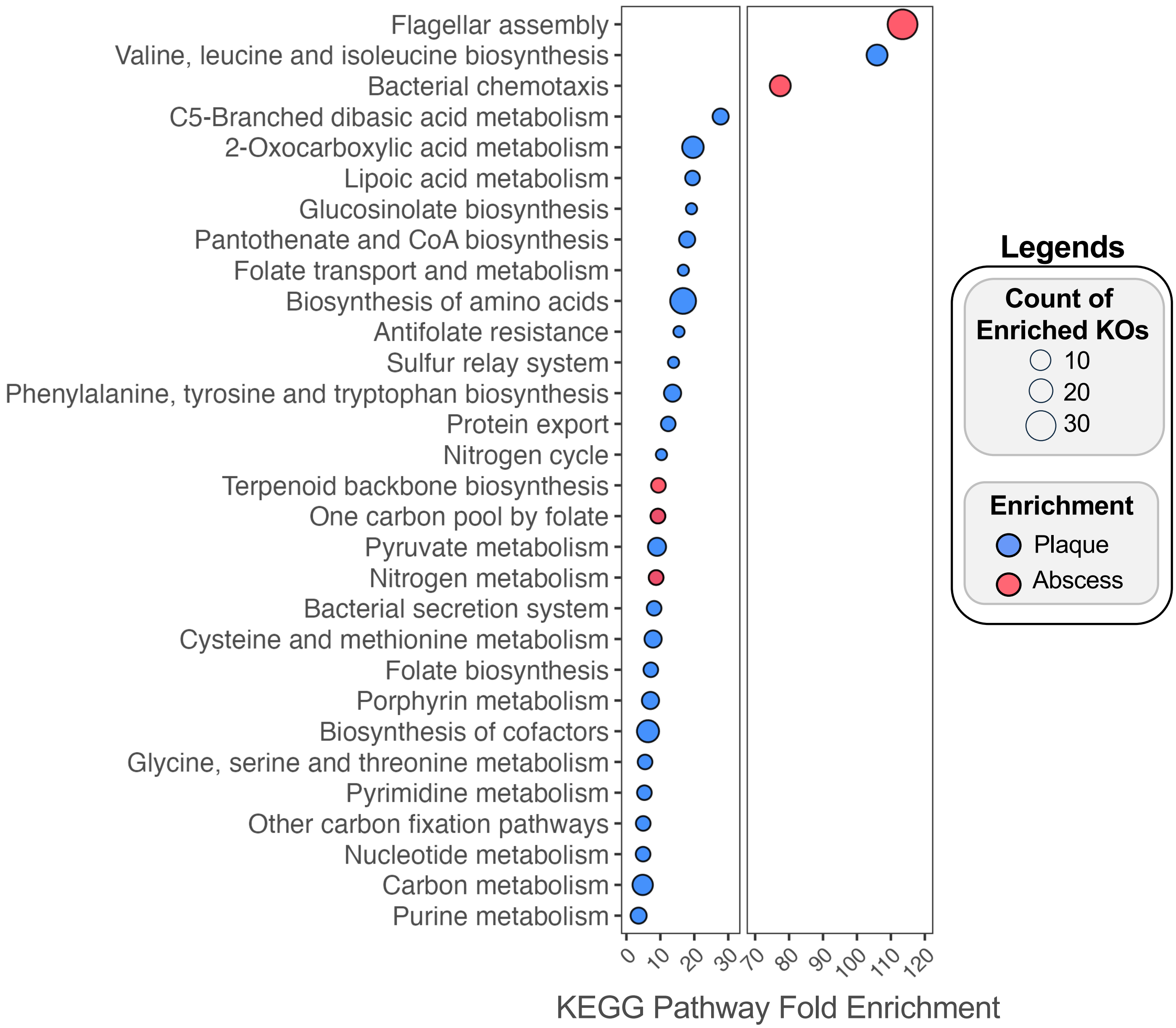

Flagellar assembly
Valine, leucine and isoleucine biosynthesis
Bacterial chemotaxis
C5-Branched dibasic acid metabolism
2-Oxocarboxylic acid metabolism
Lipoic acid metabolism
Glucosinolate biosynthesis
Pantothenate and CoA biosynthesis
Folate transport and metabolism
Biosynthesis of amino acids
Antifolate resistance
Sulfur relay system
Phenylalanine, tyrosine and tryptophan biosynthesis
Protein export
Nitrogen cycle
Terpenoid backbone biosynthesis
One carbon pool by folate
Pyruvate metabolism
Nitrogen metabolism
Bacterial secretion system
Cysteine and methionine metabolism
Folate biosynthesis
Porphyrin metabolism
Biosynthesis of cofactors
Glycine, serine and threonine metabolism
Pyrimidine metabolism
Other carbon fixation pathways
Nucleotide metabolism
Carbon metabolism
Purine metabolism
0
10
20
30
70
80
90
100
110
120
KEGG Pathway Fold Enrichment
Legends
Count of Enriched KOs
10
20
30
Enrichment
Plaque
Abscess

Figure S10
