# Supplementary material for: Paired oral clinical specimens reveal the underlying ecology supporting the emergence of inflammophilic microbiome communities
Source: Microbiol Spectr. 2026 Sep 14;14(10):e01550-26. doi: 10.1128/spectrum.01550-26 (PMC13689325; doi:10.1128/spectrum.01550-26)
Supplement: Supplementary file 3 [file spectrum.01550-26-s0003.docx]

**Supplemental Figure Legends**

**Figure S1. Species-level ecological composition of plaque and abscess communities.** (A) Principal coordinate analysis (PCoA) of species relative abundance values using Bray–Curtis distance with silhouette scores shown alongside the PCoA plot. (B) Hierarchical clustering (Pearson correlation) of log-transformed relative abundances of species separates plaque and abscess communities.

**Figure S2. Trends in high and medium confidence differentially abundant genera**. Per-sample dot plots of the relative abundance values for selected genera identified as (A) high confidence and (B) medium confidence from **Figure 4** show stable trends across the entire cohort. Genera identified as being enriched in the abscess are labeled in red, while those enriched in the plaque are labeled in blue.

**Figure S3. *Fusobacterium* abundance measured at different taxonomic levels.** Relative abundances of fusobacteria are shown at the (A) phylum, (B) genus, and species levels for (C) *Fusobacterium nucleatum* and (D) *Fusobacterium periodonticum*. Total sum scaling (TSS) values are displayed on the left of each panel, with centered log-ratio (CLR) transformed values shown on the right.

**Figure S4. Comparison of predictive accuracy for genus- and species-level sequence data.** In contrast to the random forest model built from genus-level data (**Fig. 6**), sequence data agglomerated at the species-level was less useful for classifying community type. (A) Receiver operating characteristic (ROC) curves from 4-fold cross-validation exhibit strong performance (area under the curve, or AUC values of 0.56–0.85). (C) Heatmap of feature importance rankings across folds, with top predictors in purple and lower-ranked features in green. The rank of feature importance is shown inside each cell.

**Figure S5. Algorithmic validation metrics used to select the optimum number of topics for Latent Dirichlet Allocation (LDA) topic-modeling.** Four different metrics were used to validate the performance of an LDA model used for (A) species-level and (B) genus-level data. To preserve interoperability of the model, the number of topics minimized CaoJuan2009 [89] and Arun2010 [90] metrics and maximized Deveaud2014 [91] and Griffiths2004 [92] metrics. The number of topics selected is shown with a red dotted vertical line.

**Figure S6. Patterns of species-level enrichment across six topics.** Relative abundance log values of the top taxa identified as significant drivers of each topic signature. Topics 2 and 4 (blue text) are significantly enriched in plaque specimens and topic 3 (red text) is significantly enriched in abscess communities.

**Figure S7. Importance scores of species-level topics that are shared equally between plaque and abscess specimens.** The importance (beta) scores of the top species driving topics 1, 5 and 6 are shown. These topics were not significantly enriched in either plaque or abscess communities but instead describe shared signatures of both oral communities.

**Figure S8. Topic modeling of genus-level community signatures in plaque and abscess specimens**. (A) Boxplots of sample membership (gamma values), with the results of a Wilcoxon Signed-rank test displayed above (*** = p < .001, * = p < .05). (B) Heatmap of gamma scores across topics. (C) The importance (beta) scores of the top species driving each topic. Topics 1, 4 and 6 were enriched in plaque samples, while topics 3 and 5 were enriched in abscess communities. Topics 2 and 7 described a shared microbiome signature across both plaque and abscess specimens.

**Figure S9. Patterns of genus-level taxa enrichment across seven topics.** Log values of the relative abundances of the top taxa identified as significant drivers of each topic signature. Topics 1, 4 and 6 (blue text) are significantly enriched across plaque specimens and topics 3 and 5 (red text) are significantly enriched in abscess specimens.

**Figure S10. Additional confirmation of KEGG pathways within plaque and abscess communities.** A functional enrichment analysis was performed on a pangenome constructed of enriched taxa identified in the plaque and abscess specimens. KOs that were differentially enriched either plaque and abscess samples (adjusted q value < .05) were used as inputs to identify key KEGG pathways of plaque (blue) and abscess (red) communities. The x-axis displays fold enrichment of the KEGG pathway. Dot size corresponds to the number of KOs that were identified as being part of each respective KEGG pathway (y-axis).

**Supplemental Table Legends**

**Table S1. Genus-level differences in mean and median values between plaque and abscess communities.**

**Table S2. Counts of patients having enrichment of taxa in either the plaque or abscess specimens at the genus- and species-levels.**

**Table S3. Species-level differences in mean and median values between plaque and abscess communities.**

**Table S4. Differential abundance results from three analytical methods.**

**Table S5. Counts of patients harboring enriched taxa identified by differential abundance analysis of plaque and abscess specimens.**

**Table S6. MaAsLin2 analysis of KEGG Orthologs identified by PiCRUSt2.**

**Table S7. Enriched KEGG Pathways in plaque and abscess samples identified by PiCRUSt2.**

**Table S8. Genomes used for Anvi’o functional enrichment analysis.**

**Table S9. Complete results from Anvi’o functional enrichment analysis of KEGG Orthologs.**

**Table S10. Enriched KEGG Pathways in plaque and abscess samples identified by Anvi’o.**

**Table S11. Comparison of differentially abundant taxa identified using paired versus unpaired differential abundance analysis methods.**

**Table S12. Contaminant taxa removed from analysis.**
